# Hypoxia promotes airway differentiation in the human lung epithelium

**DOI:** 10.1016/j.stem.2025.09.007

**Published:** 2025-10-10

**Authors:** Ziqi Dong, Niek Wit, Aastha Agarwal, Adam James Reid, Dnyanesh Dubal, Sina Beier, Krishnaa T. Mahbubani, Kourosh Saeb-Parsy, Jelle van den Ameele, James A. Nathan, Emma L. Rawlins

**Affiliations:** 1 Wellcome Trust/CRUK Gurdon Institute, University of Cambridge, Cambridge CB2 1QN, UK; 2 Department of Physiology, Development and Neuroscience, University of Cambridge, Cambridge CB2 3DY, UK; 3 Cambridge Institute of Therapeutic Immunology & Infectious Disease (CITIID), Jeffrey Cheah Biomedical Centre, Department of Medicine, University of Cambridge, Cambridge CB2 0AW, UK; 4 Department of Clinical Neurosciences and MRC Mitochondrial Biology Unit, University of Cambridge, Cambridge CB2 0XY, UK; 5 Department of Surgery, University of Cambridge, Cambridge NIHR Biomedical Research Centre, Cambridge CB2 0QQ, UK

## Abstract

Human lungs experience dynamic oxygen tension during development. Here, we show that hypoxia directly regulates human lung epithelial cell identity using tissue-derived organoids. Fetal multipotent lung epithelial progenitors remain undifferentiated in a self-renewing culture condition under normoxia but spontaneously differentiate toward multiple airway cell types and inhibit alveolar differentiation under hypoxia. Using chemical and genetic tools, we demonstrate that hypoxia-induced airway differentiation depends on hypoxiainducible factor (HIF) activity, with HIF1*α* and HIF2*α* differentially regulating progenitor fate decisions. KLF4 and KLF5 are direct HIF targets that promote basal and secretory cell fates. Moreover, hypoxia is sufficient to convert alveolar type 2 cells derived from both human fetal and adult lungs to airway cells, including aberrant basal-like cells that exist in human fibrotic lungs. These findings reveal roles for hypoxia and HIF activity in the developing human lung epithelium and have implications for aberrant cell fate changes in pathological lungs.

## Graphical abstract

**Graphic abstract:**
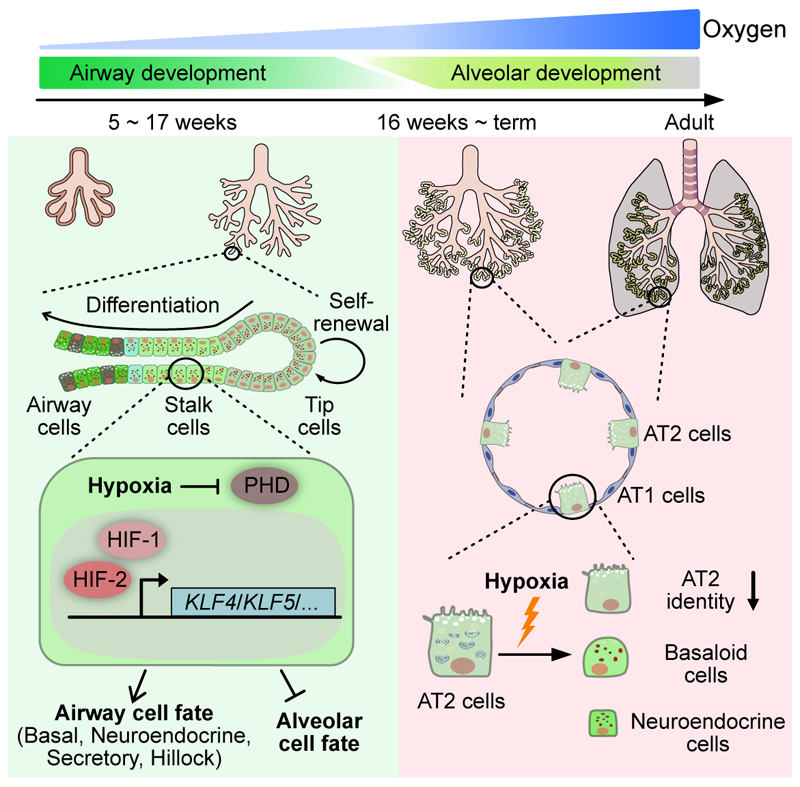

## Introduction

Human lung development starts at ~5 post-conception weeks (pcw).^1,2^ During the branching period (5–17 pcw), multipotent lung epithelial progenitors self-renew in distal tip regions (known as tip cells), initiate differentiation in adjacent stalk regions (stalk cells), and subsequently differentiate to airway epithelial cells, establishing a proximal-distal gradient of differentiation.^3–8^ The progenitors later switch to alveolar epithelial cell fate from ~ 16 pcw, with alveolar maturation extending to postnatal stages.^7–10^ Human lungs experience dynamic oxygen tension during gestation correlating with placental development, as the maternal-placental blood circulation remains unestablished until the end of the first trimester.^11,12^ Consequently, oxygen tension can be as low as ~1%–5% within first-trimester placentas.^13^ The lungs are exposed to air postnatally when alveolar oxygen tension reaches ~14%.^14^ Therefore, the human airway epithelium differentiates in a more hypoxic environment than the alveolar epithelium. We hypothesized that oxygen tension directly influences lung epithelial cell fate.

Hypoxia and hypoxia-inducible factor (HIF) activity can modulate lung development, repair, and disease.^15,16^ The activity of HIFs is primarily regulated by HIFα subunit stabilization. Briefly, in normoxia, HIFα is hydroxylated by prolyl hydroxylase domain (PHD) enzymes, then ubiquitinated by the von Hippel-Lindau (vHL) E3 ligase complex followed by proteasome degradation.^17–19^ When oxygen is limited, intact HIFα heterodimerizes with HIF1β (ARNT, aryl hydrocarbon receptor nuclear translocator) and binds hypoxia-response elements (HREs) to activate downstream genes.^20^ Hypoxia can induce *Drosophila* larva tracheal sprouting through Sima (HIFα homolog)^21,22^ and affect branching of mouse embryonic lung explants.^23,24^ Hypoxia can also induce neuroendocrine or goblet cell differentiation in the mouse or human adult lungs, respectively.^25,26^ HIF1α and HIF2α are expressed in the first-trimester human lung epithelium, though their functions in this stage remain elusive.^27^

We have used tissue-derived human lung organoids to elucidate the direct effects of hypoxia on epithelial differentiation. The epithelial progenitors derived from first-trimester lung buds spontaneously differentiated into airway cells under hypoxia, simultaneously repressing alveolar-lineage commitment. We systematically dissected the functions of HIF1α and HIF2α in regulating progenitor fate decisions and identified direct downstream targets, including KLF4 and KLF5 (KLF transcription factor 4 and 5). Furthermore, human alveolar type 2 (AT2) cells derived from second-trimester and adult lungs differentiated into airway cells under hypoxia, including aberrant basal-like cells existing in human fibrotic lungs. Therefore, hypoxia emerges as a developmental cue directly promoting airway differentiation of fetal lung epithelial progenitors, with implications for aberrant cell identity changes in disease.

## Results

### Hypoxia induces airway differentiation of human fetal lung epithelial progenitors

**Figure 1:**
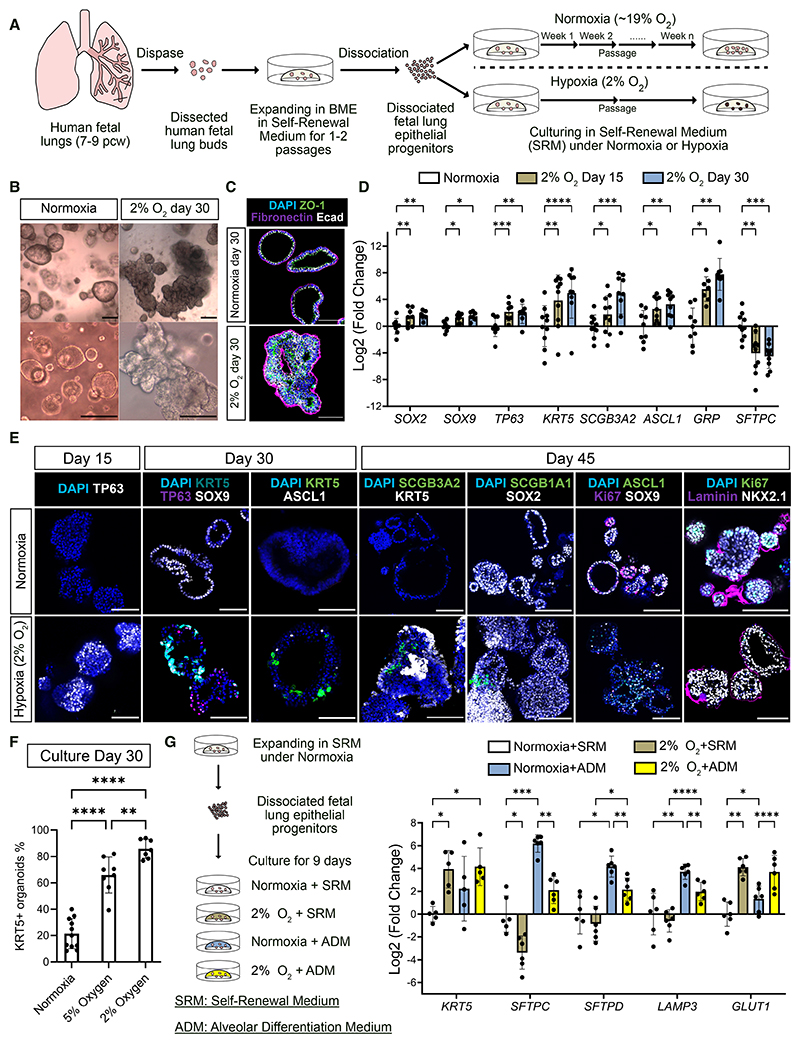
Hypoxia promotes airway differentiation of first-trimester human lung epithelial progenitors (A) Experimental design for the derivation and normoxic/hypoxic culture of lung epithelial progenitors. (B) Bright-field images of lung progenitor organoids under normoxia or hypoxia for 30 days. (C) Hypoxia-induced organoid shape changes visualized by zonula occludens-1 (ZO-1), fibronectin, and E-cadherin (Ecad). (D) Progenitor and differentiation marker gene expression under normoxia or hypoxia (2% O_2_) for 15 and 30 days, detected by RT-qPCR. Fold changes were normalized to the mean of the normoxia samples. Bars represent mean log_2_(fold change) ± standard deviation (SD), *n* = 10 experimental replicates from 8 biological donors. Statistical test: two-way ANOVA with Geisser-Greenhouse correction and Dunnett’s multiple comparisons test. (E) Immunostaining for progenitor (SOX9 and SOX2), basal (TP63 and KRT5), secretory (SCGB3A2 and SCGB1A1), and neuroendocrine (ASCL1) cells, as well as proliferation (Ki67), lung identity (NKX2.1), and extracellular matrix (laminin). DAPI: nuclei. Representative images from 4 organoid lines. (F) Percentage of organoids containing ≥1 KRT5^+^ cell(s). Average values were calculated from multiple independent experiments, *n* = 11 (normoxia), 8 (5% O_2_), and 7 (2% O_2_) from 3 biological donors. Data shown as mean ± SD. Statistical test: one-way ANOVA with Tukey’s multiple comparisons test. (G) RT-qPCR of organoids cultured in normoxia + SRM, normoxia + ADM, hypoxia + SRM, and hypoxia + ADM conditions for 9 days. Fold changes were normalized to the mean of normoxia + SRM condition. Bars represent mean log_2_(fold change) ± SD, *n* = 6 biological donors. Statistical test: two-way ANOVA with Tukey’s multiple comparisons test. Scale bars, 100 μm in all panels. Gene expression was normalized to *ACTB* in RT-qPCR. Significance levels: *p < 0.05, **p < 0.01, ***p < 0.001, ****p < 0.0001. See also Figure S1.

The fetal lung buds (7–9 pcw) containing tip and stalk cells were dissected, dissociated, and expanded as organoids under normoxia in a self-renewal medium (SRM) for 1–2 passages, while residual mesenchymal cells diminished as reported.^7,28^ We then cultured the epithelial progenitors under normoxia (~20% O_2_) or hypoxia (2% O_2_) in SRM (Figure 1A). Organoids in normoxia maintained progenitor identity across multiple passages (Figure S1A). Whereas organoids in hypoxia acquired more folded morphologies (Figures 1B and 1C), and elevated expression of airway cell markers, including basal (*TP63, Tumor Protein P63;* and *KRT5, Keratin 5*), secretory (*SCGB3A2, Secretoglobin Family 3A Member 2*), and neuroendocrine (*ASCL1, Achaete-Scute Family bHLH Transcription Factor 1*; and *GRP, Gastrin Releasing Peptide*) cells, they downregulated a canonical AT2 marker (*SFTPC, Surfactant Protein C*) (Figures 1D and 1E). Most cells lost SOX9 (SRY-Box Transcription Factor 9, tip marker) but retained SOX2 (SRY-Box Transcritption Factor 2, stalk and airway marker) (Figure 1E). Hypoxia also decreased cell proliferation (Ki67, Marker Of Proliferation Ki-67) without altering lung cell identity (NKX2.1, NK2 Homeobox 1) (Figure 1E). The organoids exposed to 5% O_2_ still upregulated airway markers and downregulated AT2 cell markers but to a lesser extent than at 2% O_2_ (Figures S1B and S1C). The percentage of organoids containing KRT5^+^ cells was also higher at 2% O_2_ than at 5% O_2_ (Figure 1F). In subsequent experiments, we used 2% O_2_ to mimic the hypoxic environment during first-trimester lung development.

To test hypoxia effects on alveolar differentiation, we cultured epithelial progenitors in either SRM or a published alveolar differentiation medium (ADM) under normoxia or hypoxia (Figure 1G).^9^ In SRM, 9-day hypoxia was sufficient to induce *KRT5* and the hypoxia-responsive gene *GLUT1* (known as *SCL2A1, Solute Carrier Family 2 Member 1*), while decreasing *SFTPC*. The ADM condition promoted AT2 cell markers (*SFTPC, SFTPD Surfactant Protein D*, and *LAMP3 Lysosomal Associated Membrane Protein 3*) under normoxia, though the effect was diminished by hypoxia (Figure 1G). We evaluated the cell state in a human fetal lung epithelial cell atlas using a hypoxia hallmark gene set.^29^ The basal and secretory cells *in vivo* had higher hypoxia scores than AT2 and AT1 (Alveolar Type 1) cells (Figure S1D). The total hypoxia scores increased during the airway formation stage (9–15 pcw) and later decreased (Figure S1E). Therefore, hypoxia-induced airway differentiation correlates with the hypoxic cell state *in vivo*.

We isolated mouse lung epithelial progenitors from the branching lung buds (E11.5–E14.5) and cultured them in a self-renewing condition.^30^ Hypoxia (2% O_2_, 6–24 days) promoted mouse airway marker genes (*Scgb1a1 Secretoglobin Family 1A Member 1, Foxj1 Forkhead Box J1*, and *Sox2*) but did not significantly change *Krt5* or *Sftpc* (Figure S1F), showing non-identical effects compared with human lung epithelial progenitors.

### Emergence of basal, neuroendocrine, secretory-like, and hillock-like cells under hypoxia

**Figure 2:**
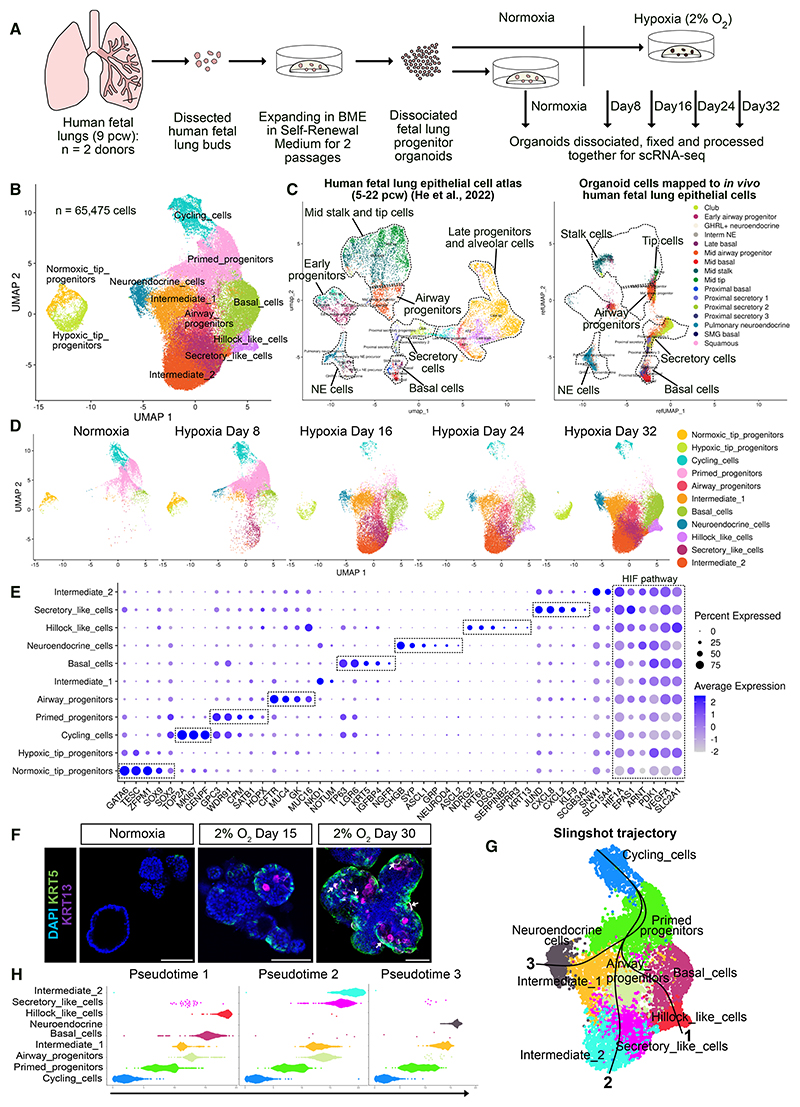
Emergence of basal, neuroendocrine, secretory-like, and hillock-like cells under hypoxia (A) Experimental design. Epithelial progenitor organoids derived from two human fetal (9 pcw) lungs were treated with normoxia or hypoxia (2% O_2_) in SRM. Organoids were sampled, dissociated, and fixed over multiple days, and processed together for library preparation. (B) UMAP of cells from all samples with annotated cell populations. (C) Left: reference UMAP of human fetal lung epithelial cell atlas. Right: all cells from the organoids projected onto the reference UMAP. (D) Organoid cells sampled at different days shown in the same UMAP. (E) Expression patterns of canonical *in vivo* lineage markers, cell-type-specific markers newly identified from the organoid dataset, and HIF pathway-related genes. (F) Hillock-like cells (KRT13^+^) emerged at days 15 and 30 in hypoxic organoids. Arrows indicate KRT5^+^KRT13^+^ cells. Scale bars, 100 μm. (G and H) Slingshot trajectory analysis. (G) Three pseudotime trajectories originating from cycling cells and diverging at primed progenitors. (H) Cell-type changes along the trajectories. See also Figures S2 and S3.

To determine the cellular dynamics underlying hypoxia-induced progenitor differentiation, we conducted a time-series single-cell RNA sequencing (scRNA-seq) experiment. We sampled organoids cultured under normoxia and 8–32 days of hypoxia from two fetal lungs (9 pcw) and processed samples together to minimize batch effects (Figure 2A). Combining all samples yielded a 65,475-cell transcriptomic dataset with >4,200 median genes per cell (Figure S2A). Overall, we identified 11 cell populations: three populations of progenitors (designated as tip, primed, and airway progenitors), differentiated airway cells (basal, neuroendocrine, secretory-like, and hillock-like cells), cycling cells, and two intermediate populations (Figure 2B). The two donor replicates were highly consistent, as visualized by uniform manifold approximation and projection (UMAP) (Figure S2B), and had similar contributions to most cell types (Figure S2C). We therefore merged data from both organoid lines for analysis.

By benchmarking against *in vivo* human fetal lung epithelial cells,^8^ the organoid cells were mainly mapped to mid-stage (9–11 pcw) tip and stalk cells, airway progenitors, and differentiated airway cells (basal, neuroendocrine, and secretory cells) but not to late-stage (15–22 pcw) progenitors or alveolar cells (Figure 2C). The cycling cells in organoids had mixed identities and persisted under hypoxia, consistent with the slow expansion phenotype of hypoxic organoids (Figures 2D and S2D–S2F).

The increased gene capture rate compared with previous scRNA-seq experiments revealed unexpected heterogeneity in the normoxic self-renewing organoids (Figure 2D).^8^ Normoxic organoids consisted of two progenitor populations, cycling cells, and small proportions of differentiating cells (Figures 2D and S2G). We designated the progenitor populations as “tip” and “primed” progenitors, as *in vitro* counterparts of tip and stalk cells that represent different states of lung epithelial progenitors *in vivo*. Tip progenitors were *SOX9*^high^*SOX2*^low^, highly expressed tip cell markers *GATA6* (*GATA Binding Protein 6*) and *TESC* (*Tescalcin*) (Figures 2E and S2H), and had strong regulon activity for SOX9 and GATA6 (Figure S3A).^5,7,8,31^ Primed progenitors were *SOX9*^low^*SOX2*^high^*TESC*^−^, with a subset expressing *HOPX* (*HOP Homeobox*), a stalk cell marker (Figures 2E and S2H).^8^ Primed progenitors expressed a progenitor surface marker (*CPM, Carboxypeptidase M*) and *TP63*, with high FOXA1 (Forkhead Box A1) regulon activity, indicating a differentiation-primed state (Figures 2E and S3A).^32^ In a human fetal lung Xenium transcriptomics dataset,^33^ tip cells, stalk cells, and differentiating airway cells were spatially clustered in a distal-proximal gradient (Figure S3B). Tip cells were marked by *TESC, SOX9, SFTPC*, and *LGR5* (*Leucine Rich Repeat Containing G Protein-Coupled Receptor 5*), whereas stalk cells expressed higher levels of *SOX2, CPM*, and differentiation genes (*TP63, ASCL1, SCGB3A2*, and *SCGB1A1*) (Figures S3C and S3D). We further confirmed that TESC protein was expressed in tip cells but not in stalk cells, and the existence of both TESC^+^ and TESC^−^ organoids derived from fetal lung buds (Figure S3E). Therefore, the SRM condition maintained both undifferentiated tip cells and differentiation-primed stalk cells under normoxia.

Most cell populations that predominated in hypoxia could be assigned to known *in vivo* cell types. Airway progenitors highly expressed *CFTR* (*CF Transmembrane Conductance Regulator*), *MUC4* (*Mucin 4 Cell Surface Associated*), and *MUC16* (*Mucin 16 Cell Surface Associated*), the airway precursor and secretory cell markers.^8,33,34^ Basal (*TP63, KRT5, LGR6 Leucine Rich Repeat Containing G Protein-Coupled Receptor 6*, and *NGFR Nerve Growth Factor Receptor*) and neuroendocrine (*ASCL1, GRP, CHGB Chromogranin B, SYP Synaptophysin, ASCL2 Achaete-Scute Family bHLH transcription Factor 2*, and *NEUROD4 Neuronal Differentiation 4*) cells expressed canonical markers (Figures 2E and S2H). Surprisingly, we identified a hillock-like cell population from hypoxia day 16, marked by *KRT6A Keratin 6A, KRT13 Keratin 13, DSG3 Desmoglein 3, SERPINB2 Serpin Family B Member 2*, and *SPRR3 Small Proline Rich Protein 3* (Figures 2D, 2E, and S2H).^35,36^ Some KRT13^+^ cells co-expressed KRT5 (Figure 2F). The secretory-like cells expressed *SCGB3A2* and chemokine genes (*CXCL8 C-X-C Motif Chemokine Ligand 8* and *CXCL2 C-X-C Motif Chemokine Ligand 2*) that are enriched in proximal secretory cells *in vivo*.^8^ Secretory-like cells also highly expressed stress-responsive gene *JUND* (*Jun Proto-Oncogene AP-1 Transcription Factor Subunit*) with activated c-Jun N-terminal kinase (JNK) and nuclear factor κB (NF-κB) pathways (Figures 2E, S2H, and S3A).

To infer relationships between different cell populations, we conducted trajectory analysis using Slingshot.^37^ The trajectory starts from cycling cells and branches at primed progenitors (Figure 2G). One branch leads to basal and hillock-like cells, and the second branch leads to airway progenitors, secretory-like cells, and intermediate-2 cells. The third branch leads to neuroendocrine cells through intermediate-1 cells (Figures 2G and 2H). This branching trajectory matched the actual emergence order of cell populations (Figure 2D) and the Monocle 3 trajectory (Figures S3F and S3G),^38^ suggesting that primed progenitors undergo these fate decisions. In contrast, the tip progenitors in hypoxia downregulated cell cycle genes without expressing differentiation markers (Figures 2E, S3H, and S3I).

### The HIF pathway is activated under hypoxia and sufficient to drive progenitor differentiation

**Figure 3:**
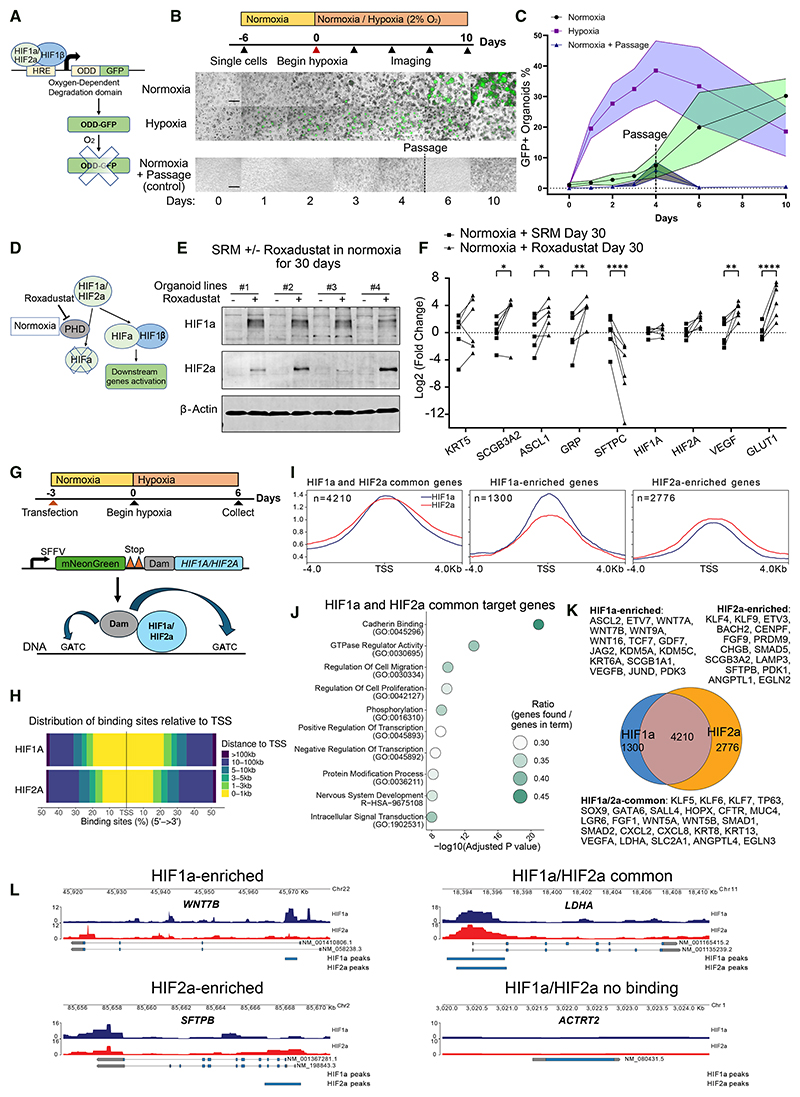
The HIF pathway is activated in hypoxic lung organoids (A) Diagram of HRE-ODD-GFP reporter. (B) Microscope images of merged bright-field and GFP channels. The progenitors with HRE-ODD-GFP reporter were cultured under normoxia for 6 days to form organoids, then treated with normoxia or hypoxia for 10 days without passaging. Control cells were cultured under normoxia with routine passaging. Scale bars, 600 μm. (C) The percentage of organoids containing ≥1 GFP^+^ cell(s). Data shown as mean ± SD, *n* = 7 (normoxia), and 8 (hypoxia) experimental replicates from 2 biological donors. (D) Roxadustat (FG-4592) inhibits PHD enzymes under normoxia and stabilizes HIFα subunits. (E) HIF1α and HIF2α were stabilized under normoxia by Roxadustat in 4 organoid lines with β-actin as loading control. (F) Roxadustat treatment activated the HIF pathway under normoxia and recapitulated hypoxia-induced airway differentiation. RT-qPCR detection of organoids cultured in SRM ± Roxadustat for 30 days. Fold changes were normalized to the mean of SRM - Roxadustat (with DMSO) condition. Data shown as log_2_(fold change), *n* = 6 biological donors. Statistical test: two-way ANOVA with Bonferroni’s multiple comparisons test. (G) Design of targeted DamID-seq for HIF1α and HIF2α. Dam-HIFα fusion proteins are expressed at a low level due to rare translation reinitiation events. The fusion proteins methylate adenines in the GATC sequences near their DNA-binding sites. (H) Global distribution of HIF1α- and HIF2α-binding sites relative to the transcription start site (TSS). (I) Quantification of HIF1α- and HIF2α-binding signals surrounding the TSS. HIF1α and HIF2α signals were normalized to Dam-only control. (J) Gene ontology analysis of HIF1a and HIF2a common target genes. (K) Venn diagram comparing HIF1α and HIF2α target genes with highlighted gene lists. Complete gene lists in Tables S1 and S2. (L) Gene track views showing averaged DamID signals and consensus peaks from three biological replicates over representative genes. Gene expression was normalized to *ACTB* in RT-qPCR. Significance levels: *p < 0.05, **p < 0.01, ***p < 0.001, ****p < 0.0001. See also Figure S4 and Tables S1 and S2.

Canonical HIF-pathway genes (*PDK1 Pyruvate Dehydrogenase Kinase 1, VEGFA Vascular Endothelial Growth Factor A*, and *SLC2A1/GLUT1*) were activated under hypoxia (Figure 2E). To monitor HIF activity, we used the HRE-ODD-GFP reporter construct.^39^ The oxygen-dependent degradation (ODD)-domain-tagged GFP is expressed when stabilized HIFs bind to the HRE under hypoxia and is rapidly degraded when oxygen tension increases (Figure 3A). Under normoxia, frequent passaging (routine laboratory practice) maintains GFP^+^ organoids (with ≥1 GFP^+^ cells) at baseline levels. However, GFP^+^ organoids accumulated when organoids were not passaged, potentially due to insufficient oxygen diffusion and increased oxygen consumption (Figures 3B and 3C). In contrast, GFP was rapidly activated upon hypoxia exposure. The GFP^+^ organoid proportion peaked at day 4 and then gradually decreased, suggesting temporal regulation of HIF activity (Figures 3B and 3C). Stabilized HIF1α and HIF2α were detected under hypoxia (Figure S4A). Moreover, the expression of HIF-target genes (*VEGFA* and *GLUT1*), *HIF2A*, and *KRT5* increased within 1 week of hypoxia culture (Figure S4B).

To test whether activation of the HIF pathway alone was sufficient to drive airway differentiation, we applied Roxadustat (FG-4592), a PHD inhibitor (Figure 3D). Both HIF1α and HIF2α were stabilized by Roxadustat under normoxia (Figure 3E). Roxadustat supplementation in SRM increased airway markers (*KRT5, SCGB3A2, ASCL1*, and *GRP*) and HIF-target genes (*VEGFA* and *GLUT1*) but decreased *SFTPC* (Figure 3F). These results supported that hypoxia-induced airway differentiation was mediated by HIFs.

### Targeted DamID-seq maps the genomic binding sites of HIF1*α* and HIF2*α*

As HIF1α and HIF2α were both stabilized by hypoxia and Roxadustat, we used targeted DNA adenine methyltransferase identification (DamID) sequencing to distinguish their genomic binding sites.^40,41^ Organoids derived from three fetal lungs were transduced by Dam-only control, Dam-*HIF1A*, or Dam-*HIF2A* fusion constructs and treated with hypoxia for 6 days (Figure 3G). The Dam control samples were clustered together, while Dam-HIF1α and Dam-HIF2α partially overlapped along principal components (Figure S4C). We normalized Dam-HIF1α and Dam-HIF2α signals to Dam control across the genome to calculate enrichment levels of HIF1α and HIF2α binding. We defined consensus peaks only if the peaks existed in all three biological replicates. HIF1α and HIF2α consensus peaks were mostly enriched in transcription start site (TSS) or promoter-adjacent regions (Figures 3H and S4D). We identified HIF1α/HIF2α target genes by assigning consensus peaks to the nearest TSS (Figure 3I).

To analyze overall HIF activity *in vivo*, we combined HIF1α and HIF2α targets and analyzed their expression in a Visium dataset of human fetal lungs.^31^ Different subsets of HIF-target genes were enriched in the epithelia of 8-, 9-, and 10-pcw lungs (Figure S4E; Table S1). The commonly expressed 337 genes were involved in glycolysis, lung morphogenesis, neuron differentiation, and tight junctions (Figure S4E). In the previously described Xenium dataset (Figures S3B–S3D), tip and stalk cells differentially enriched HIF-target genes related to cell cycle, lipid metabolism, signal transduction, and differentiation (Figures S4F and S4G). Consistently, the accessible chromatin of tip and stalk cells enriched different subsets of HIF targets (Figure S4H).^8^ These data indicate that HIFs are active *in vivo* during epithelial branching and have distinct functions in tip and stalk cells.

In the organoids, HIF1α and HIF2α shared 4,210 target genes involved in cell migration, proliferation, transcription, and protein modification (Figures 3I–3K; Table S2). The HIF1α-enriched (1,300 genes) and HIF2α-enriched (2,776 genes) target genes included diverse lineage markers and signaling pathways (Figures 3K, 3L, and S4I), suggesting that HIF1α and HIF2α have both common and distinct functions in hypoxic lung organoids.

### HIF1*α* is required for hypoxia-induced airway differentiation

**Figure 4:**
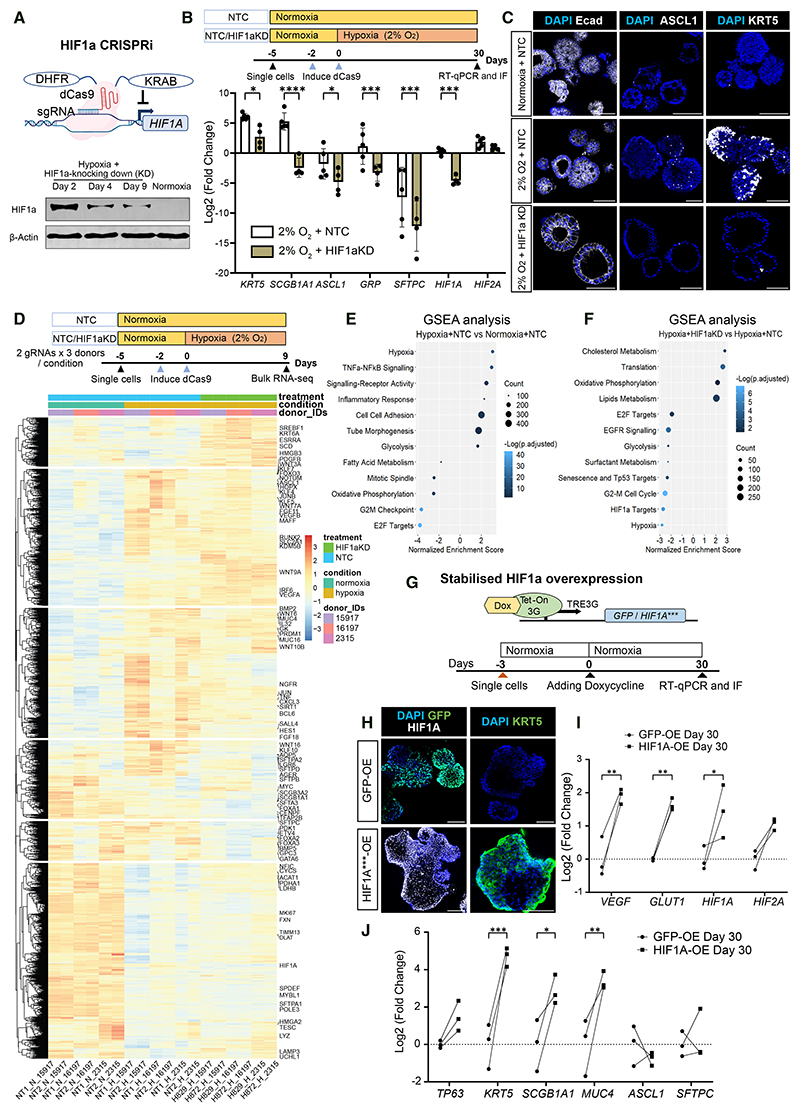
HIF1*α* is required for hypoxia-induced airway differentiation (A) HIF1α is inhibited by CRISPRi. The dCas9-KRAB effector tagged with DHFR (dihydrofolate reductase) was stabilized in the presence of trimethoprim (TMP) to reduce leaky expression from the Tet-on promoter. HIF1α protein levels decreased after 4–9 days knockdown (KD) under hypoxia as shown by western blot. (B) Upper: experimental design. NTC or *HIF1A*-KD organoids were cultured under normoxia or hypoxia for 30 days. The dCas9 was induced at −2 days by doxycyline and TMP. Lower: RT-qPCR results. Fold changes were normalized to the mean of NTC + normoxia condition (not shown). Bars represent mean log_2_(fold change) ± SD, *n* = 4 experimental replicates from 3 biological donors. 2 gRNAs tested. (C) Immunostaining of organoids with NTC or *HIF1A*-KD induction for 30 days showed changes in organoid shape (Ecad) and differentiation (ASCL1 and KRT5). Representative images from 3 organoid lines. (D) The NTC and *HIF1A*-KD organoids were cultured under normoxia or hypoxia for 9 days and used for bulk RNA-seq with 2 gRNAs and 3 biological donors for each condition. Heatmap showing DEGs (|log_2_(fold change)| > 0.5, *p*adj < 0.05, merged from DEGs in comparisons of hypoxia + NTC vs. normoxia + NTC, and hypoxia + HIF1αKD vs. hypoxia + NTC) across all samples, with representative genes labeled. (E and F) GSEA results of 9,621 DEGs (*p*adj < 0.05) between hypoxia + NTC vs. normoxia + NTC (E), 5,904 DEGs (*p*adj < 0.05) between hypoxia + HIF1αKD vs. hypoxia + NTC (F). Complete DEGs and GSEA results listed in Table S3. (G) Stabilized form of HIF1α was induced by Tet-On system under normoxia with GFP as control. (H) Immunostaining of organoids overexpressing GFP or HIF1α for 30 days under normoxia. Representative images of 2 organoid lines. (I and J) HIF1α overexpression under normoxia induced HIF pathway genes (I) and differentiation genes (J). Fold changes were normalized to the mean of GFP-overexpression organoids. Data shown as log_2_(fold change), *n* = 3 biological donors. Scale bars, 100 μm. For RT-qPCR, Gene expression was normalized to *ACTB*. Statistical test: two-way ANOVA with Bonferroni’s multiple comparisons test. Significance levels: *p < 0.05, **p < 0.01, ***p < 0.001, ****p < 0.0001. See also Figure S5 and Table S3.

We used an inducible CRISPRi system to interrogate HIF1α and HIF2α functions.^42^ The CRISPRi system efficiently knocked down HIF1α using previously evaluated gRNAs (Figure 4A).^43^ To examine how depleting HIF1α affects progenitor differentiation, we cultured non-targeting control (NTC) or *HIF1A*-targeting gRNA (guide RNA)-transduced organoids under hypoxia (2% O_2_) for 30 days. Inhibition of *HIF1A* limited the hypoxia-induced expression of airway markers (*KRT5, SCGB1A1, ASCL1*, and *GRP*). Intriguingly, HIF1α knockdown resulted in even lower *SFTPC* expression than NTC (Figures 4B and 4C).

To delineate the primary effects of hypoxia and HIF1α knockdown, we cultured NTC and *HIF1A*-targeting organoids under either normoxia or hypoxia for 9 days (Figure 4D). The NTC organoids upregulated airway markers and downregulated *SFTPC* under hypoxia, and the airway gene expression was efficiently rescued by knocking down *HIF1A* to ~10% of control levels (Figure S5A). We performed bulk RNA-seq using two different NTC/*HIF1A* gRNAs and three biological donors for each condition at day 9 (Figure 4D). Comparing NTC organoids between hypoxia and normoxia resulted in 9,621 differentially expressed genes (DEGs) (*p*adj < 0.05; Table S3). Gene set enrichment analysis (GSEA) revealed that the DEGs induced by hypoxia were associated with hypoxia response, glycolysis, cell-cell adhesion, and inflammatory response (Figure 4E). The hypoxia-responsive genes included fibroblast growth factor (FGF), Wingless and Int-1 (WNT), epidermal growth factor (EGF), and VEGF signaling pathways and transcription factors associated with developmental processes, such as *KLF4, KLF5, HOPX*, and *ASCL1*. Conversely, genes related to cell cycle, oxidative phosphorylation, and fatty acid metabolism were downregulated under hypoxia (Figures 4E and S5B).

Comparing *HIF1A*-targeting and NTC organoids under hypoxia resulted in 5,904 DEGs (*p*adj < 0.05; Table S3). The downregulated genes in *HIF1A*-targeting organoids involved hypoxia response, glycolysis, cell cycle, TP53 targets, and EGFR signaling (Figures 4F and S5C). Suppressing *HIF1A* decreased primed progenitor markers (*GPC3 Glypican 3, WDR91 WD Repeat Domain 91, CPM, HOPX*, and *FOXA1*) but not tip progenitor markers (*TESC* and *SOX9*) (Figure 4D). Interestingly, surfactant protein genes (*SFTPA1/2 Surfactant Protein A1/2, SFTPC, SFTPD Surfactant Protein D*, and *SFTA3 Surfactant Associated 3*) and other AT2 (*LYZ, Lysozyme*; and *NAPSA, Napsin A Aspartic Peptidase*) and AT1 (*AQP5, Aquaporin 5*; and *AGER, Advanced Glycosylation End-Product Specific Receptor*) cell markers also decreased after *HIF1A* depletion (Figures 4D and S5C), suggesting HIF1α may function to maintain alveolar gene expression under hypoxia. Conversely, HIF1α inhibition upregulated genes involved in oxidative phosphorylation as well as lipid and cholesterol metabolism (Figures 4F and S5C).

To determine whether inducing HIF1α alone is sufficient to drive progenitor differentiation, we used the doxycycline (Dox)-inducible TetON system to overexpress stabilized HIF1α (Figure 4G). The stabilized HIF1α carries three point mutations on hydroxylation sites (P402A, P564A, and N803A) to prevent its degradation or the suppression of its transactivation functions under normoxia.^44^ Stabilized HIF1α was detected in nuclei of normoxic organoids, along with widespread KRT5^+^ cells (Figure 4H). Overexpressing HIF1α increased *VEGFA* and *GLUT1* expression compared with control GFP overexpression, though *HIF2A* also increased 2-fold (Figure 4I). Basal (*KRT5*), secretory (*SCGB1A1*), and airway progenitor (*MUC4*) cell markers were activated by HIF1α over-expression, whereas neuroendocrine (*ASCL1*) and AT2 (*SFTPC*) cell markers remained unaffected (Figure 4J). We previously reported an airway differentiation medium (AWDM) to derive basal and secretory cells from lung progenitors under normoxia.^8^ Knocking down *HIF1A* did not affect airway differentiation in AWDM (Figure S5D), suggesting that hypoxic and chemical signaling act independently to promote airway differentiation.

### HIF2*α* promotes basal but inhibits secretory, neuroendocrine, and alveolar cell fates

**Figure 5:**
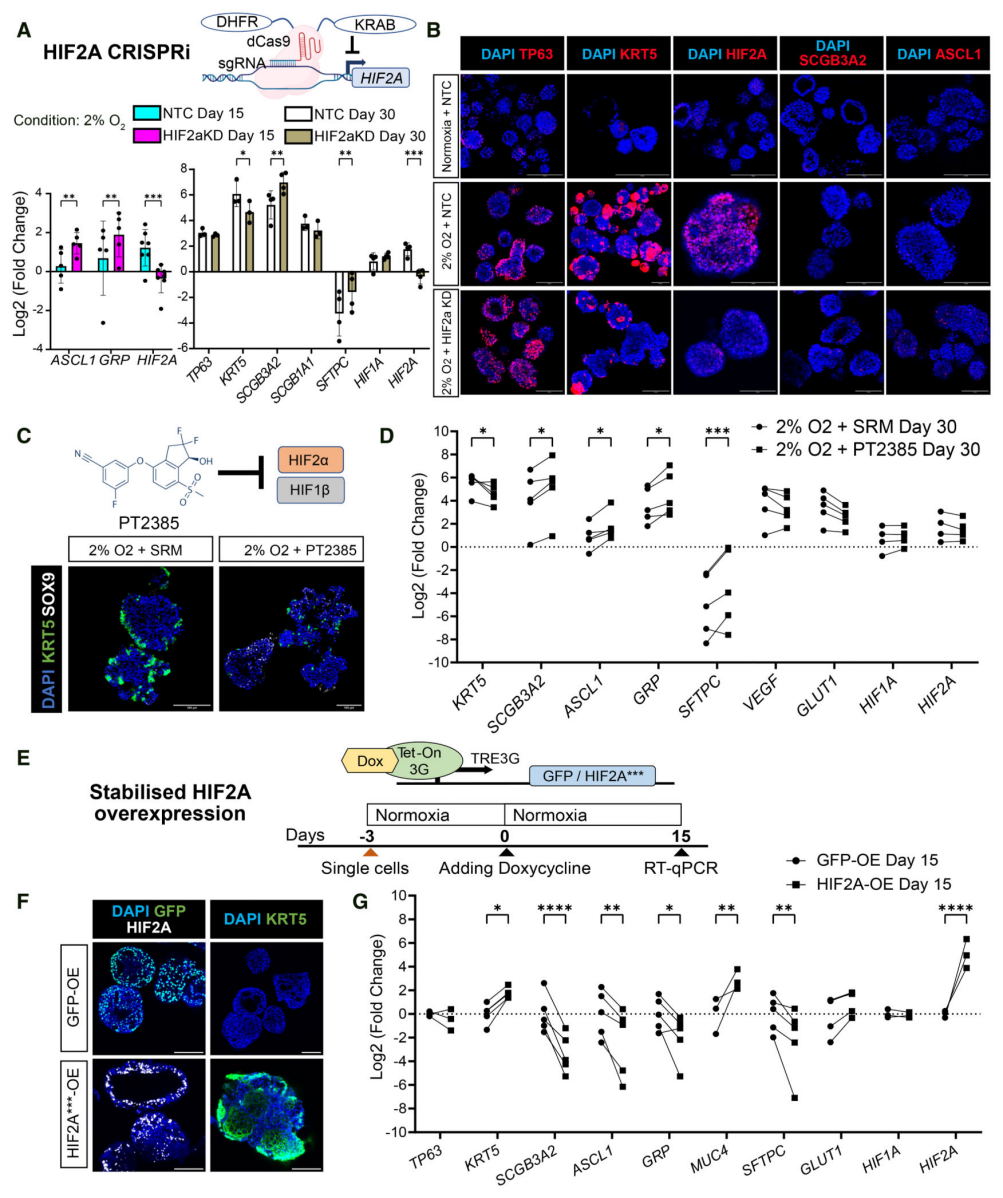
HIF2α promotes basal, but inhibits secretory, neuroendocrine, and alveolar, cell fates (A) RT-qPCR results of NTC and *HIF2A*-KD organoids cultured under hypoxia for 15 and 30 days. Fold changes were normalized to the mean of NTC + normoxia condition (not shown). Bars represent mean log2(fold change) ± SD, *n* = 5 (day 15) and 4 (day 30) experimental replicates from 3 biological donors. 2 gRNAs used. (B) Immunostaining of organoids with NTC or *HIF2A*-KD induction for 30 days. Representative images of 2 organoid lines. (C) PT2385 inhibits heterodimerization between HIF2α and HIF1β. Treatment with PT2385 under hypoxia for 30 days decreased KRT5+ cells while increasing SOX9+ cells. Representative images of 2 organoid lines. (D) RT-qPCR from organoids treated with SRM only (with DMSO) or SRM + PT2385 under hypoxia for 30 days. Fold changes were normalized to the mean of normoxia + SRM condition (not shown). Data shown as log2(fold change), *n* = 5 biological donors. (E) Stabilized form of HIF2α was induced by Tet-On system under normoxia, with GFP-overexpression as control. (F) Immunostaining of organoids overexpressing GFP or HIF2α for 15 days under normoxia. Representative images of 2 organoid lines. (G) RT-qPCR from HIF2α overexpression under normoxia for 15 days. Fold changes were normalized to the mean of GFP-overexpression organoids. Data shown as log_2_(fold change), *n* = 5 experimental replicates from 4 biological donors. Scale bars, 100 μm. For RT-qPCR, gene expression was normalized to *ACTB*. Statistical test: two-way ANOVA with Bonferroni’s multiple comparisons test. Significance levels: *p < 0.05, **p < 0.01, ***p < 0.001, ****p < 0.0001.

To determine the role of HIF2α in regulating progenitor differentiation, we used CRISPRi to knock down *HIF2A*. Suppressing *HIF2A* under hypoxia inhibited a basal cell marker (*KRT5*) but promoted neuroendocrine (*ASCL1* and *GRP*), secretory (*SCGB3A2*), and AT2 (*SFTPC*) cell markers (Figure 5A). More SCGB3A2^+^ and ASCL1^+^ cells, but fewer KRT5^+^ cells, appeared in *HIF2A*-targeting organoids than NTC organoids under hypoxia (Figure 5B). Consistent with *HIF2A*-CRISPRi results, treatment of a selective HIF-2 antagonist, PT2385, under hypoxia reduced KRT5^+^ cells (Figure 5C) and decreased *KRT5* but promoted *SCGB3A2, ASCL1, GRP*, and *SFTPC* gene expression (Figure 5D). Overexpressing a stabilized form of HIF2α with three point mutations (P405A, P531A, and N847A)^44^ under normoxia promoted basal cell differentiation but inhibited secretory, neuroendocrine, and AT2 marker expression (Figures 5E–5G). These results demonstrated distinct functions between HIF2α and HIF1α in mediating lung progenitor differentiation.

### KLF4 and KLF5 promote basal and secretory cell fates downstream of HIFs

**Figure 6:**
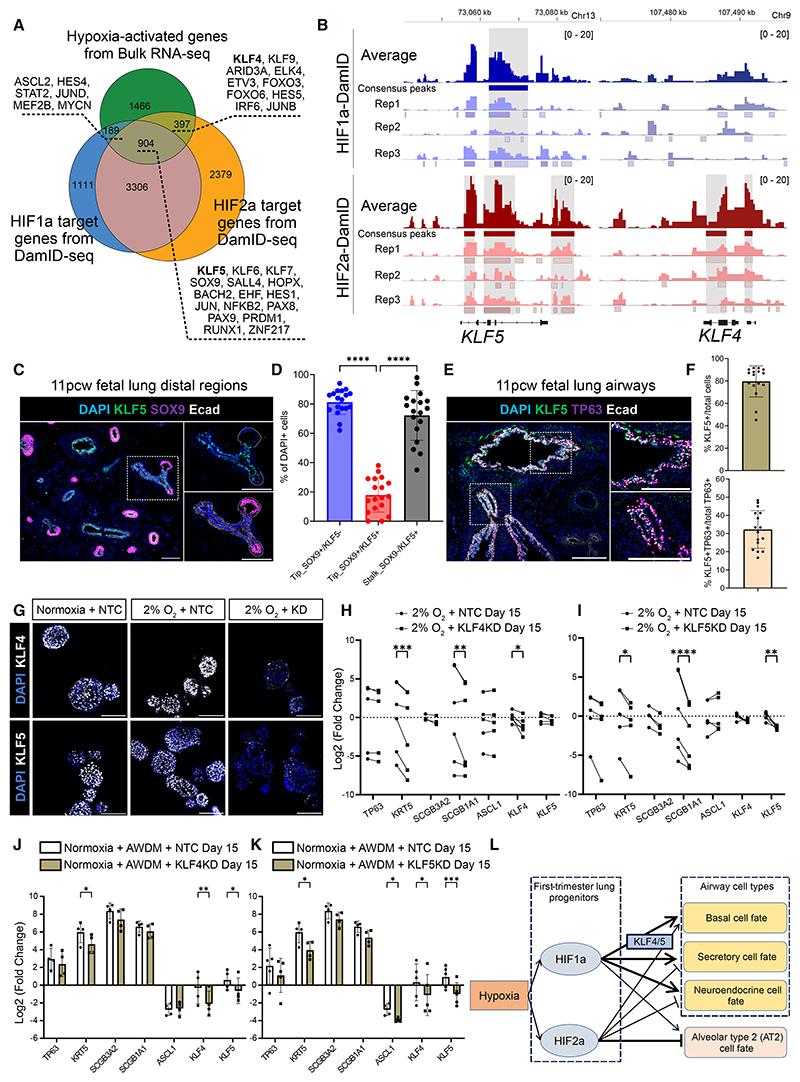
KLF4 and KLF5 promote basal and secretory cell fates downstream of the HIF pathway (A) Venn diagram of HIF1α- and HIF2α-binding genes from targeted DamID-seq (false discovery rate [FDR] < 0.01) and hypoxia-activated genes from bulk RNA-seq (log_2_(fold change) > 0.5, *p*adj < 0.05, DEGs of hypoxia + NTC vs. normoxia + NTC). Complete gene lists in Table S4. (B) Gene track views showing respective and averaged DamID signals from three biological replicates over *KLF4* and *KLF5* with consensus peaks labeled. (C–F) Immunostaining and quantification of 11 pcw human fetal lung sections showing KLF5, epithelium (Ecad), tip cells (SOX9), and differentiating basal cells (TP63) in distal regions (C and D) and airways (E and F). Quantification with lung sections from 3 donors. Statistical test: one-way ANOVA with Bonferroni’s multiple comparisons test. ****p < 0.0001. (G) Immunostaining of organoids with NTC, *KLF4*-KD (upper), or *KLF5*-KD (lower) by CRISPRi induction for 15 days. Representative images of 2 organoid lines for each gene. (H and I) RT-qPCR results of *KLF4*-KD (H) and *KLF5*-KD (I) compared with NTC in SRM under hypoxia for 15 days. Data shown as log_2_(fold change), *n* = 6 experimental replicates from 4 (*KLF4*) and 5 (*KLF5*) biological donors. 2 gRNAs used for each gene. (J and K) RT-qPCR results of *KLF4*-KD (J) and *KLF5*-KD (K) compared with NTC in AWDM under normoxia for 15 days. Data shown as log_2_(fold change), *n* = 4 (*KLF4*), 5 (*KLF5*) experimental replicates from 2 (*KLF4*) and 3 (*KLF5*) biological donors. 2 gRNAs used for each gene. (L) Proposed mechanisms underlying hypoxia-induced airway differentiation. Scale bars, 100 μm. RT-qPCR gene expression was normalized to *ACTB*. Statistical test: two-way ANOVA with Bonferroni’s multiple comparisons test. Significance levels: *p < 0.05, **p < 0.01, ***p < 0.001, ****p < 0.0001. See also Figure S6 and Table S4.

Combining HIF-binding genes from DamID-seq and hypoxia-activated genes from bulk RNA-seq, we predicted primary targets of HIF1α and HIF2α (Figure 6A; Table S4). Many development-related transcription factors were identified, such as *KLF5* in HIF1α/HIF2α common targets (904 genes), *ASCL2* in HIF1α targets (189 genes), and *KLF4* in HIF2α targets (397 genes) (Figures 6A and 6B). Manipulating HIF1α and HIF2α differentially regulated *KLF4* and *KLF5* expression (Figures S6A–S6E). In the organoid scRNA-seq dataset, *KLF4* was enriched in hypoxia-induced cell types while *KLF5* was more ubiquitous (Figure S6F). In a human fetal lung atlas,^8^ both *KLF4* and *KLF5* are expressed in airway progenitors and differentiated airway cells (Figure S6G). In the spatial transcriptomic analysis, stalk cells had higher *KLF5* expression than tip cells (Figure S3D). In firsttrimester human fetal lungs, <20% of SOX9^+^ tip cells co-expressed KLF5, while ~70% of stalk cells were KLF5^+^ (Figures 6C and 6D). In the proximal airway epithelium, ~80% of epithelial cells were KLF5^+^ and ~30% of TP63^+^ cells co-expressed KLF5 (Figures 6E and 6F). In contrast, KLF4 was expressed more broadly throughout the fetal lung, including in mesenchymal cells (Figure S6H).

We tested the hypothesis that KLF4 and KLF5 regulate airway differentiation downstream of HIFs using CRISPRi. KLF4 and KLF5 were expressed in NTC organoids under normoxia and hypoxia but depleted in CRISPRi organoids (Figure 6G). *KLF4*-CRISPRi and *KLF5*-CRISPRi both limited basal (*KRT5*) and secretory (*SCGB1A1*) cell markers but not *ASCL1* under hypoxia (Figures 6H and 6I). *KLF4*-CRISPRi did not affect AT2 cell markers, whereas *KLF5*-CRISPRi decreased *SFTPD* and *SLC34A2* (Figures S6I and S6J). Therefore, KLF4 and KLF5 both mediated basal and secretory cell differentiation but differentially affected AT2 cell fate under hypoxia.

To determine whether KLF4 and KLF5 are also required for biochemical-induced airway differentiation, we cultured *KLF4*-CRISPRi and *KLF5*-CRISPRi organoids in AWDM under normoxia. *KLF4* and *KLF5* inhibition resulted in lower levels of basal and secretory cell differentiation (Figures 6J and 6K). *KLF5* inhibition also decreased *ASCL1* expression in this condition. Interestingly, inhibiting either *KLF4* or *KLF5* decreased the other’s expression. These data suggested that hypoxia and chemical signaling potentially converged through KLF4/KLF5 to promote basal and secretory cell fates.

Taken together, we propose a working model to explain the roles of HIF1α and HIF2α in regulating progenitor fate decisions under hypoxia (Figure 6L). HIF1α is required for airway differentiation and maintaining alveolar programs, whereas HIF2α promotes basal cell fate but inhibits other airway and, especially, alveolar cell fates. KLF4 and KLF5 are direct HIF targets mediating basal and secretory cell differentiation.

### Chronic hypoxia drives human AT2-to-airway cell differentiation

**Figure 7:**
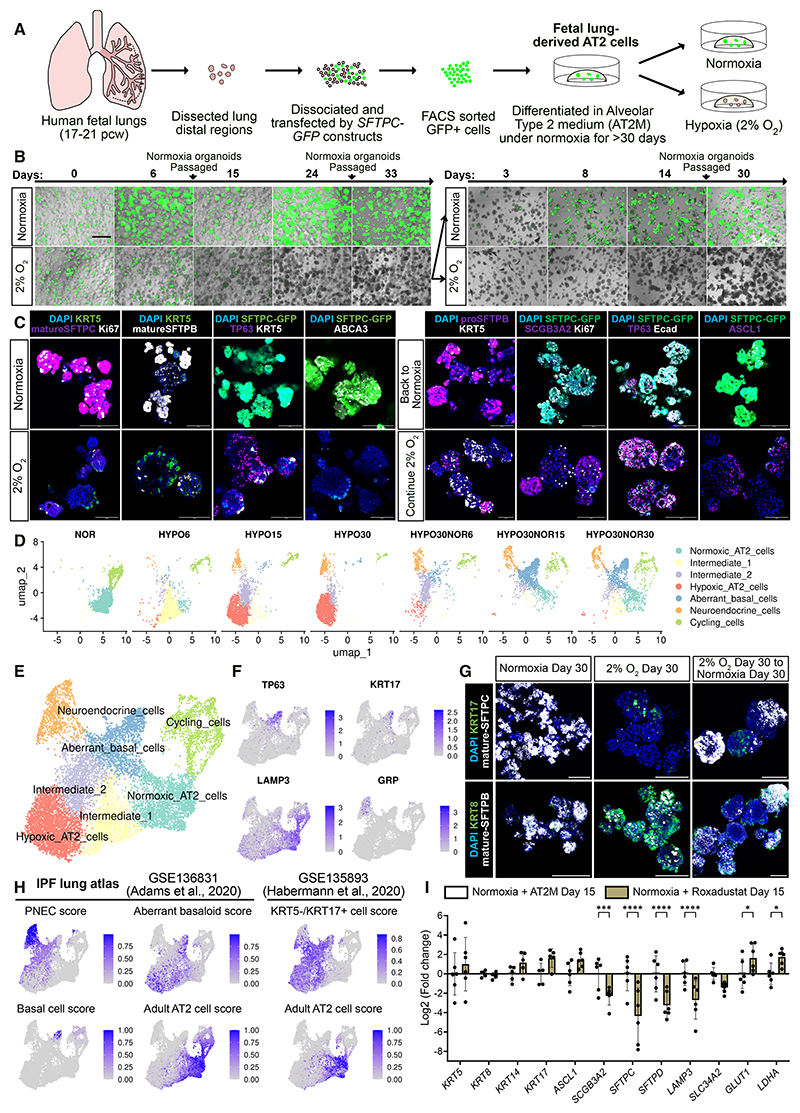
Chronic hypoxia converts human AT2 cells to airway cells (A) Experimental design. Distal epithelial cells were isolated from second-trimester human fetal (17–21 pcw) lungs, transduced by *SFTPC-GFP* reporter, fluorescence-activated cell sorting (FACS) sorted, and cultured in AT2M as fdAT2 organoids under normoxia or hypoxia. (B) Images of merged bright-field and GFP channels. The fdAT2 organoids with *SFTPC-GFP* reporter were cultured under normoxia (with passaging) or hypoxia (without passaging) for 33 days. The hypoxia-treated organoids were split and cultured under normoxia or hypoxia for another 30 days. Scale bars, 600 μm. Representative images of 2 organoid lines. (C) Immunostaining of AT2 and airway cell markers of fdAT2 organoids cultured under normoxia, hypoxia (30 and 60 days), and re-exposure to normoxia (30 days). Representative images of 3 organoid lines. Scale bars, 100 μm. (D) UMAP of scRNA-seq data from fdAT2 organoids at each time point. (E) Cell cluster annotation of the fdAT2 organoid scRNA-seq dataset. (F) Feature plots showing *TP63, KRT17, LAMP3*, and *GRP*. (G) Immunostaining of fdAT2 organoids cultured under normoxia, hypoxia (30 days), and re-exposure to normoxia (30 days). Representative images of 2 organoid lines. Scale bars, 100 μm. (H) Cell-type prediction scores. The fdAT2 organoid dataset was projected onto two human IPF lung atlases. Cell-type names for scoring were retrieved from the respective atlases. (I) RT-qPCR of fdAT2 organoids treated with Roxadustat under normoxia for 15 days. Fold changes were normalized to the mean of fdAT2 without Roxadustat treatment (with DMSO). Data shown as mean log_2_(fold change) ± SD, *n* = 6 experimental replicates from 4 biological donors. Gene expression was normalized to *ACTB*. Significance levels: *p < 0.05, ***p < 0.001, ****p < 0.0001. See also Figures S7 and S8.

As hypoxia suppressed alveolar fate in first-trimester lung epithelial progenitors (Figure 1G), we investigated how differentiated AT2 cells responded to hypoxia using fetal-lung-derived AT2 (fdAT2) organoids.^45^ The fdAT2 cells recapitulate mature AT2 cell features, including surfactant protein production, lamellar body formation, and AT1 cell differentiation.^45^ We isolated and transduced alveolar-fated distal epithelial cells from second-trimester lungs (17–21 pcw) with an *SFTPC-GFP* reporter construct, sorted GFP^+^ cells, and differentiated them in AT2 medium (AT2M) to obtain fdAT2 cells (Figure 7A). Under normoxia, the fdAT2 cells proliferated and maintained *SFTPC*-GFP expression (Figure 7B). In contrast, the fdAT2 cells decreased *SFTPC*-GFP levels within 1 week of hypoxia treatment (Figure 7B). The fdAT2 cells lost canonical AT2 markers (mature-SFTPB, mature-SFTPC, and ABCA3 ATP Binding Cassette Subfamily A Member 3), reduced proliferation (Ki67), and acquired basal cell markers (TP63 and KRT5) (Figure 7C). Hypoxia-treated fdAT2 cells also showed time-dependent downregulation of AT2 markers and upregulation of HIF targets (Figure S7A).

To determine whether hypoxia effects were reversible, we split the day-33 hypoxia-treated fdAT2 organoids into either normoxia or hypoxia and cultured for a further 30 days. *SFTPC*-GFP^+^ cells re-appeared within 3 days of returning to normoxia and gradually increased (Figure 7B). Moreover, the organoids regained pro-SFTPB, recovered proliferation (Ki67), and mostly lost airway markers, though some cells remained TP63^+^ or ASCL1^+^ (Figures 7C and S7B). In comparison, the organoids kept in hypoxia remained negative for AT2 markers and positive for airway markers (Figure 7C).

We performed time-series scRNA-seq for one fdAT2 organoid line (Figures 7D, 7E, and S7C). The initial fdAT2 organoids contained only normoxic AT2 cells and cycling cells. However, the majority of AT2 cells transitioned through an intermediate state and entered a hypoxic state (hypoxic AT2 cells) under hypoxia. Re-exposure to normoxia partially recovered the normoxic AT2 cell population (Figures 7D, S7D, and S7E). Neuroendocrine cells appeared from hypoxia day 15 and were retained after returning to normoxia. Interestingly, a *TP63*^+^*KRT5*^−^ cell population (aberrant basal cells) emerged under hypoxia and expanded extensively following re-oxygenation (Figures 7D and S7E). The *TP63*^+^ cells expressed *KRT8* (*Keratin 8*), *KRT15* (*Keratin 15*), *KRT17* (*Keratin 17*), and *GPR87* (*G Protein-Coupled Receptor 87*), markers of basaloid cells in human fibrotic lungs (Figures 7F and S7F).^46–48^ *KRT5*^+^ cells were not represented, potentially due to limited sample size. KRT17^+^ and KRT8^+^ cells were confirmed by immunostaining in hypoxia-treated fdAT2 organoids (Figure 7G). We mapped this dataset to two human adult lung atlases containing fibrotic samples.^46,47^ The normoxic AT2 cells showed high similarity to adult AT2 cells in both atlases. The neuroendocrine cells and a subset of aberrant basal cells in organoids showed high accordance with pulmonary neuroendocrine cells (PNECs) and basal cells, respectively. In contrast, the intermediate-state cells, hypoxic AT2 cells, and aberrant basal cells scored highly for the basaloid or KRT5^−^/KRT17^+^ cell signatures (Figure 7H). Compared with normoxic AT2 cells, aberrant basal cells activated epithelial-mesenchymal transition, Notch signaling, and hypoxia response, the previously reported basaloid cell signatures (Figure S7G).^47,49,50^ The hypoxic AT2 cells instead activated mTORC1 (mammalian target of rapamycin complex 1) signaling, unfolded protein response, glycolysis, and tRNA aminoacylation (Figure S7H). These data suggest that hypoxia was sufficient to activate an airway differentiation program in fdAT2 cells, including the emergence of disease-state basaloid cells.

We investigated the role of HIFs using pharmacological approaches. Activating HIF signaling under normoxia using Roxadustat consistently downregulated AT2 markers and non-significantly upregulated airway markers (*KRT5, KRT14 Keratin 14, KRT17*, and *ASCL1*) (Figure 7I). In contrast, inhibiting HIF-2 using PT2385 increased AT2 but not airway markers (Figure S7I). The loss of AT2 cell identity in hypoxia was potentially mediated by HIF-2.

We further examined the hypoxia effect using adult-lung-derived AT2 (adAT2) cells. We cultured HTII-280^+^ epithelial cells isolated from adult human lung distal parenchyma in AT2M or a reported serum-free, feeder-free (SFFF) medium (Figures S8A and S8B).^51^ Compared with SFFF medium, AT2M increased *ASCL1* but did not significantly change AT2 markers (Figure S8C). The adAT2 cells cultured in AT2M under hypoxia decreased *SFTPC, SFTPD*, and *ASCL1* but increased *KRT14* and *KRT17* (Figure S8D). The adAT2 cells lost mature SFTPC/SFTPB while KRT5^+^ cells emerged (Figure S8E). To check reversibility of hypoxia effects, we cultured adAT2 cells in SFFF medium under hypoxia (2% O_2_, 15 days) and return to normoxia (15 days). The adAT2 cells again downregulated AT2 markers (*SFTPC, SFTPD*, and *LAMP3*) and activated airway genes (*KRT14, KRT17*, and *ASCL1*) under hypoxia, while normoxia re-exposure efficiently rescued gene expression changes (Figure S8F). However, KRT17^+^ and KRT5^+^ cells remained in normoxia (Figure S8G). Therefore, hypoxia promotes an airway differentiation program at the expense of AT2 cell identity, even in adult lungs.

## Discussion

The human lung epithelium exhibits a stronger hypoxic signature during airway development than during alveolar development. Consistent with this, first-trimester lung epithelial progenitors autonomously differentiated into airway cells at the expense of alveolar fate under hypoxia. We showed that tip and stalk cells, which represent different progenitor states, differentially responded to hypoxia. Our analysis suggests that the differential effects may originate from intrinsic factors like chromatin structure, although cellular metabolism and local niche signals potentially also contribute.

We observed differential functions of HIF1α and HIF2α in developing human lung epithelium. HIF1α and HIF2α both promoted basal cell fate but had opposing effects on secretory, neuroendocrine, and alveolar cell fates. Similarly, Hif1α promotes, while Hif2α inhibits, the differentiation of basal cells to neuroendocrine cells in the mouse trachea.^25^ Through targeted DamID-seq, we found that HIF1α and HIF2α regulated distinct sets of target genes. The functional differences of HIF1α and HIF2α may also result from different co-factor recruitment or crosstalk with other pathways.

Beyond promoting airway differentiation, HIF signaling is critically involved in alveolar development. Insufficient alveolar maturation can lead to respiratory distress syndrome (RDS) in newborn infants. In rodent models, Hif1α in the alveolar epithelium is essential for AT2 cell maturation and surfactant generation.^52^ In contrast, Hif2α is required for Vegf-mediated blood vessel maturation, while its overexpression in the epithelium leads to RDS.^53–55^ Our results demonstrate that, in human lung progenitors, HIF1α is required for maintaining the expression of surfactant synthesis genes under hypoxia, whereas HIF2α consistently inhibits AT2 cell fate. As the models in this study have only epithelial cells, the functions of HIF signaling in non-epithelial cells and their indirect effects on epithelial development await further investigation.

Local hypoxia can occur in adult lungs during injury and chronic disease. In influenza-infected mouse lungs, Hif1α promotes ectopic basal cell growth from the airways into the alveolar epithelium.^56,57^ In mouse fibrotic lungs, Hif2α inhibition attenuates fibrosis and promotes alveolar regeneration.^50^ These results are consistent with the roles of HIF1α and HIF2α in the developing human lung epithelium. In human fibrotic lungs, aberrant basaloid cells can accumulate in alveolar regions and exhibit hypoxic signatures.^46–50,58^ Human AT2 cells have been shown to transdifferentiate to basal cells through a basaloid-like cell state when cocultured with pathogenic mesenchyme.^58^ Our results indicate that human AT2 cells can directly sense hypoxia and give rise to neuroendocrine cells and aberrant basal cells *in vitro*. In our experiments, HIF signaling was directly responsible for loss of the AT2 cell identity and activation of the airway differentiation program. Future studies deciphering the downstream effects of HIFs could improve our understanding of hypoxia-induced lung remodeling and facilitate discovery of intervention targets.

### Limitations of the study

The experiments in this study utilized well-characterized and highly reductionist systems that stably maintain the identity of lung epithelial progenitors and AT2 cells *in vitro*. However, one technical limitation is that the cells were exposed to normoxia during isolation and the initial expansion phase, which differs from the physiological oxygen level. Additionally, the cell and organoid culture conditions were established under an ambient oxygen environment. To develop more physiologically relevant *in vitro* models, it will be important to control oxygen tension from the setup of the system in future. The organoids in this study contain only epithelial cells, enabling detailed investigation of the direct effects of hypoxia on lung epithelial cells. However, lung development and maintenance rely on complex interactions among multiple cell types within specialized niches. Therefore, it will also be important to examine the effects of hypoxia on other lung cell types and intercellular interactions using more complex *in vitro* and *in vivo* models.

## Resource Availability

### Lead contact

Requests for further information and resources should be directed to, and will be fulfilled by, the lead contact, Emma Rawlins (elr21@cam.ac.uk).

### Materials availability

This study did not generate new unique reagents.

## Star★Methods

### Key Resources Table

**Table:** 

REAGENT or RESOURCE	SOURCE	IDENTIFIER
Antibodies		
Rabbit anti-KLF4	Proteintech	Cat# 11880-1-AP; RRID: AB_10640807
Rabbit anti-KLF5	Proteintech	Cat# 21017-1-AP; RRID: AB_10696447
Rabbit anti-SOX9	Millipore	Cat# AB5535; RRID: AB_2239761
Goat anti-SOX9	R&D Systems	Cat# AF3075; RRID: AB_2194160
Goat anti-TP63	R&D Systems	Cat# AF1916; RRID: AB_2207174
Rabbit anti-p63alpha	Cell Signaling Technology	Cat# 13109; RRID: AB_2637091
Rat anti-E-cadherin	Thermo Fisher Scientific	Cat# 13-1900; RRID: AB_2533005
Goat anti-SOX2	R&D Systems	Cat# AF2018; RRID: AB_355110
Mouse anti-HT2-280	Terrace biotech	Cat# TB-27AHT2-280; RRID: AB_2832931
Rabbit anti-mature SFTPC	Seven Hills Bioreagents	Cat# WMAB-76694; RRID: N/A
Rabbit anti-mature SFTPB	Seven Hills Bioreagents	Cat# WMAB-48604; RRID: N/A
Rabbit anti-proSFTPB	Seven Hills Bioreagents	Cat# WMAB-55522; RRID: N/A
Mouse anti-ABCA3	Seven Hills Bioreagents	Cat# WMAB-17G524; RRID: N/A
Rabbit anti-ZO-1	Thermo Fisher Scientific	Cat# 40-2200; RRID: AB_2533456
Mouse anti-KI67	BD Biosciences	Cat# 550609; RRID: AB_393778
Chicken anti-KRT5	BioLegend	Cat# 905901; RRID: AB_2565054
Rabbit anti-SCGB3A2	Abcam	Cat# ab181853; RRID: AB_2938818
Rabbit anti-SCGB1A1	Proteintech	Cat# 10490-1-AP; RRID: AB_2183285
Sheep anti-Fibronectin	R&D Systems	Cat# AF1918; RRID: AB_2105832
Rabbit anti-NKX2.1	Abcam	Cat# ab76013; RRID: AB_1310784
Rabbit anti-ASCL1	Abcam	Cat# EPR19840; RRID: N/A
Rabbit anti-Laminin	Abcam	Cat# ab11575; RRID: AB_298179
Goat anti-KRT13	Abcam	Cat# ab79279; RRID: AB_2281128
Rabbit anti-HIF-1α	Novus Biologicals	Cat# NB100-134; RRID: AB_350071
Rabbit anti-HIF-2α	Novus Biologicals	Cat# NB100-122; RRID: AB_10002593
Rabbit anti-HIF-2α	Cell Signaling Technology	Cat# 7096; RRID: AB_10898028
Mouse anti-β-Actin	Merck	Cat# A1978; RRID: N/A
Rabbit anti-TESC	Proteintech	Cat# 11125-1-AP; RRID: N/A
Mouse anti-KRT8	Santa Cruz	Cat# sc-8020; RRID: N/A
Mouse anti-KRT17	Santa Cruz	Cat# sc-393002; RRID: N/A
Donkey anti-rabbit Alexa Fluor 488	Thermo Fisher Scientific	Cat# A-21206
Donkey anti-goat Alexa Fluor 594	Thermo Fisher Scientific	Cat# A-11058
Donkey anti-mouse Alexa Fluor 488	Thermo Fisher Scientific	Cat# A-21202
Donkey anti-rat Alexa Fluor 488	Thermo Fisher Scientific	Cat# A-21208
Donkey anti-mouse Alexa Fluor 594	Thermo Fisher Scientific	Cat# A-21203
Donkey anti-rabbit Alexa Fluor 594	Thermo Fisher Scientific	Cat# A-21207
Donkey anti-rabbit Alexa Fluor 647	Thermo Fisher Scientific	Cat# A-31573
Donkey anti-mouse Alexa Fluor 647	Thermo Fisher Scientific	Cat# A-31571
Donkey anti-goat Alexa Fluor 647	Thermo Fisher Scientific	Cat# A-21447
Goat anti-mouse IgM Alexa Fluor 488	Thermo Fisher Scientific	Cat# A-21042
Donkey anti-mouse IRDye 800CW	Abcam	Cat# ab216774
Donkey anti-rabbit IRDye 680RD	Abcam	Cat# ab216779
Donkey anti-chicken Alexa Fluor 488	Jackson ImmunoResearch	Cat# 703-545-155
Donkey anti-sheep Alexa Fluor 594	Jackson ImmunoResearch	Cat# 713-585-147
Donkey anti-rat Alexa Fluor 647	Jackson ImmunoResearch	Cat# 712-605-153
Biological samples		
Fetal lung-derived organoid lines: HDBR 14580,15909,15917,16186,16197,16217, 16393,15328,16392,15350,14489, 14710, 14731, 14556, 16402, 16587	HDBR London and Newcastle	N/A
Fetal lung-derived organoid lines: BRC 1915, 1943, 2315, 2316	Brain Repair Center, University of Cambridge	N/A
Adult lung-derived AT2 organoid lines: 847, 881,887, 889, 890, 900	Cambridge Biorepository for Translational Medicine	N/A
Mouse embryonic lung epithelial progenitor organoids derived from wild-type C57BL/6J mice	Charles River Laboratories	N/A
Chemicals, peptides, and recombinant proteins		
2,20-Thiodiethanol (TDE)	Merck	Cat# 166782
PrimeSTAR GXL DNA Polymerase	Takara Bio Europe	Cat# R050A
In-Fusion HD Cloning Plus	Takara Bio Europe	Cat# 638910
BbsI-HF	New England BioLabs	Cat# R3539S
T4 DNA ligase	New England BioLabs	Cat# M0202S
T4 Polynucleotide Kinase	New England BioLabs	Cat# M0201S
Alkaline Phosphatase	New England BioLabs	Cat# M0290S
Agarose	Merck	Cat# A5304
MultiScribe Reverse Transcriptase	Thermo Fisher Scientific	Cat# 4311235
Paraformaldehyde	Sigma-Aldrich	Cat# 158127-500G
Bovine serum albumin	Sigma-Aldrich	Cat# A9647-100G
Normal donkey serum	Jackson ImmunoResearch	Cat# 017.000.121
Optimum Cutting Temperature	Tissue Tek	Cat# 4583
Triton X-100	Sigma-Aldrich	Cat# 1001246242X100
Halt Protease and Phosphatase Inhibitor Cocktail	Thermo Fisher Scientific	Cat# 78440
RIPA buffer	Merck	Cat# R0278
Cell Recovery Solution	Corning	Cat# 354253
RBC lysis buffer	BioLegend	Cat# 420301
Dispase	Thermo Fisher Scientific	Cat# 17105041
DNase	Merck	Cat# D4527
Collagenase	Merck	Cat# C9891
Trimethoprim	Merck	Cat# 92131
Doxycycline	Merck	Cat# D9891
DAPT	Merck	Cat# D5942
Dexamethasone	Merck	Cat# D4902
Y-27632	Merck	Cat# 688000
3-Isobutyl-1-methylxanthine (IBMX)	Merck	Cat# I5879
8-Bromoadenosine 3’ 5’-cyclic monophosphate (cAMP)	Merck	Cat# B5386
N-acetylcysteine	Merck	Cat# A9165
N2 supplement	Thermo Fisher Scientific	Cat# 17502001
B27 supplement	Thermo Fisher Scientific	Cat# 12587001
EGF	PeproTech	Cat# AF-100-15
FGF10	PeproTech	Cat# 100-26
FGF7	PeproTech	Cat# 100-19
Noggin	PeproTech	Cat# 120-10C
R-spondin	Cambridge Stem Cell Institute	N/A
CHIR99021	Cambridge Stem Cell Institute	N/A
SB431542	Bio-techne	Cat# 1614
A83-01	Tocris	Cat# 2939
Advanced DMEM/F12	Thermo Fisher Scientific	Cat# 12634-010
Penicillin/Streptomycin	Thermo Fisher Scientific	Cat# 15140-122
Hepes	Thermo Fisher Scientific	Cat# 15630-056
GlutaMax	Thermo Fisher Scientific	Cat# 35050-038
N2	Thermo Fisher Scientific	Cat# 17502-048
B27	Thermo Fisher Scientific	Cat# 12587-010
Insulin-Transferrin-Selenium	Thermo Fisher Scientific	Cat# 41400-045
Fgf9	R&D Systems	Cat# 7399-F9-025
Heparin	Sigma-Aldrich	Cat# H3149
BIRB796	Tocris	Cat# 5989
Basement Membrane Extract	Bio-Techne	Cat# 3533-010-02
DMSO	Sigma-Aldrich	Cat# D2650
Lipofectamine 2000	Thermo Fisher Scientific	Cat# 11668019
TrypLE Express	Thermo Fisher Scientific	Cat# 12605-010
Trypan Blue Solution	Thermo Fisher Scientific	Cat# 15250061
DreamTaq HS DNA Polymerase	Thermo Fisher Scientific	Cat# EP1703
Lenti-X Concentrator	Takara Bio Europe	Cat# 631232
CD326 (EpCAM) microbeads	Miltenyi Biotec	Cat# 130-061-101
Roxadustat (FG-4592)	Selleckchem	Cat#S1007
PT2385	Selleckchem	Cat# S8352
Fluoromount	Merck	Cat# F4680
Critical commercial assays		
QIAprep Spin Miniprep Kit	Qiagen	Cat# 27104
Qiaquick Gel Extraction Kit	Qiagen	Cat# 28704
EndoFree Plasmid Maxi Kit	Qiagen	Cat# 12362
RNeasy Plus Mini Kit	Qiagen	Cat# 74134
RNase-Free DNase Set	Qiagen	Cat# 79254
PowerUp SYBR Green Master Mix	Thermo Fisher Scientific	Cat# A25741
Pierce BCA Protein Assay Kit	Thermo Fisher Scientific	Cat# 23225
Evercode Cell Fixation kit	Parse Biosciences	N/A
Evercode Whole Transcriptome v2 kit	Parse Biosciences	N/A
Evercode Whole Transcriptome v3 kit	Parse Biosciences	N/A
Qubit dsDNA HS Assay Kit	Thermo Fisher Scientific	Cat# Q32854
NEBNext Ultra II DNA Library Prep Kit	New England BioLabs	Cat# E7645S
LookOut Mycoplasma PCR Detection Kit	Merck	Cat# MP0035
High-Capacity cDNA Reverse Transcription Kit	Thermo Fisher Scientific	Cat# 4368814
Deposited data		
Human lung epithelial progenitor organoids scRNA-seq in normoxia and hypoxia	This paper	GEO: GSE273089
HIF1a and HIF2a DamID-seq	This paper	GEO: GSE272859
Human lung epithelial progenitor organoids bulk RNA-seq for control and HIF1a CRISPRi	This paper	GEO: GSE272860
Human fetal lung-derived AT2 organoids scRNA-seq in normoxia and hypoxia	This paper	GEO: GSE296547
Spatial transcriptomic data of human fetal lungs by 10x Visium	Sountoulidis et al.^31^	GEO: GSE215897
Spatial transcriptomic data of human fetal lungs by 10X Xenium	Quach et al.^33^	GEO: GSE264425
Adult human lung scRNA-seq atlas of idiopathic pulmonary fibrosis and chronic obstructive pulmonary disease	Adams et al.^47^	GEO: GSE136831
Adult human lung scRNA-seq atlas of idiopathic pulmonary fibrosis	Habermann et al.^46^	GEO: GSE135893
Human fetal lung scRNA-seq atlas	He et al.^8^	ArrayExpress: E-MTAB-11278
Human fetal lung scATAC-seq data	He et al.^8^	ArrayExpress: E-MTAB-11266
Oligonucleotides		
gRNA-HIF1A_1: GCTGGCCGAAGCGACGAAGA	Horlbeck et al.^43^	N/A
gRNA-HIF1A_2: GCCTCCTGTCCCCTCAGACG	Horlbeck et al.^43^	N/A
gRNA-HIF2A_1: GGAGGCGGCCGTACAATCCT	Horlbeck et al.^43^	N/A
gRNA-HIF2A_2: GGGCCGCCTCAGGAGCGCTG	Horlbeck et al.^43^	N/A
gRNA-KLF4_1: GCGCGGAGCTGCGAACTGGT	Horlbeck et al.^43^	N/A
gRNA-KLF4_2: GGACTGCACCGCCCAGACAT	Horlbeck et al.^43^	N/A
gRNA-KLF5_1: GCTCTCGCGGAGGTCGGCGG	Horlbeck et al.^43^	N/A
gRNA-KLF5_2: GGTTCTCTCGCGGAGGTCGG	Horlbeck et al.^43^	N/A
Recombinant DNA		
pLenti-tetON-KRAB-dCas9-DHFR- EF1aTagRFP-2A-tet3G	Sun et al.^42^	Addgene: #167935
pLenti-U6-gRNA-EF1a-EGFP-CAAX	Sun et al.^42^	Addgene: #167936
HRE-ODD-GFP reporter	Ortmann et al.^39^	N/A
pLenti-hSPC-eGFP-EF1 a-TagRFP	Lim et al.^9^	Addgene: #201681
SFFV-mNeonGreen-Dam	Sun et al.^28^	N/A
SFFV-mNeonGreen-Dam-HIF1A	This paper	N/A
SFFV-mNeonGreen-Dam-HIF2A	This paper	N/A
pLenti-tetON-HIF1A-EF1a-TagRFP- 2A-tet3G	This paper	N/A
pLenti-tetON-HIF2A-EF1a-TagRFP- 2A-tet3G	This paper	N/A
Software and algorithms		
nf-core/rnaseq pipeline v3.9	Ewels et al.^59^	https://github.com/nf-core/rnaseq
DESeq2	Love et al.^60^	https://bioconductor.org/packages/release/bioc/html/DESeq2.html
Gene Set Enrichment Analysis	Subramanian et al.^29^	https://www.gsea-msigdb.org/gsea/index.jsp
split-pipe v1.1.1	Parse Biosciences	N/A
split-pipe v1.5.0	Parse Biosciences	N/A
Seurat v5	Hao et al.^61^	https://satijalab.org/seurat/
Monocle 3	Cao et al.^38^	https://cole-trapnell-lab.github.io/monocle3/
Slingshot	Street et al.^37^	https://bioconductor.org/packages/slingshot/
SCENIC	Aibar et al.^62^	https://github.com/aertslab/SCENIC
Enrichr	Chen et al.^63^	https://maayanlab.doud/Enrichr/
DamID-seq Snakemake workflow	Wit et al.^64^	https://doi.org/10.5281/zenodo.10737672
bowtie2 v2.5.3	Langmead et al.^65^	https://github.com/BenLangmead/bowtie2
damidseq_pipeline v1.5.3	Marshall et al.^66^	https://github.com/owenjm/damidseq_pipeline
pyGenomeTracks v3.8	Lopez-Delisle et al.^67^	https://github.com/deeptools/pyGenomeTracks
MACS2 V2.2.9.1	Feng et al.^68^	https://github.com/macs3-project/MACS/releases/tag/v2.2.9.1
ChIPseeker V1.38.0	Yu et al.^69^	https://github.com/YuLab-SMU/ChIPseeker
deepTools V3.5.4	Rami’rez et al.^70^	https://github.com/deeptools/deepTools
GraphPad Prism software v10	GraphPad Prism	https://www.graphpad.com/
Fiji V2.15.1	Schindelin et al.^71^	https://imagej.net/software/fiji/
Other		
Hypoxia incubator Galaxy 48R	New Brunswick	N/A
Sony SH800Z Cell Sorter	Sony Biotechnology	N/A
BD FACSDiscover S8 Cell Sorter	BD Biosciences	N/A
Leica SP8 confocal microscope	Leica Microsystems	N/A
Nikon AxR confocal microscope	Nikon Instruments	N/A
Agilent 4200 Tapestation	Agilent	N/A

### Experimental Model And Study Participant Details

#### Human fetal and adult lung tissue

Human embryonic and fetal lung tissues were provided from Cambridge University Hospitals NHS Foundation Trust under NHS Research Ethical Committee (96/085) and the MRC/Wellcome Trust Human Developmental Biology Resource (London and Newcastle, University College London (UCL) site REC reference: 18/LO/0822; Newcastle site REC reference: 18/NE/0290; Project 200454; www.hdbr.org). Stages of the samples were evaluated by external appearance and measurements to determine their age in postconception weeks (pcw). Human adult lung tissues were provided from Cambridge Biorepository for Translational Medicine (CBTM) (reference: 15/EE/0152). None of the samples used for this study had known genetic abnormalities.

#### Derivation and maintenance of human fetal lung epithelial progenitor organoids

Human fetal lung epithelial progenitor organoids were derived as previously reported.^7^ Briefly, human fetal lung tissues (7-9 pcw) were dissociated with Dispase (8 U/mL Thermo Fisher Scientific, 17105041) at room temperature for 2 min. Mesenchyme was removed with forceps. Branching epithelial tips were micro-dissected, transferred into basement membrane extract (BME, Bio-Techne, 3533-010-02) on 24-well suspension culture plates (M9312-100EA, Greiner). The organoids were expanded in Self-Renewal Media (SRM) consisting of AdvDMEM+++ medium [Advanced DMEM/F12 (ThermoFisher Scientific, 12634010) with 1x GlutaMax (ThermoFisher Scientific, 35050061), 10 mM HEPES (ThermoFisher Scientific, 15630056) and 100 U/mL Penicillin/Streptomycin (ThermoFisher Scientific, 15140122)] and supplements [N2 (1:100, ThermoFisher Scientific, 17502–048), B27 (1:50, ThermoFisher Scientific, 12587–010), 1.25 mM N-acetylcysteine (Merck, A9165), 5% v/v R-spondin condition medium (Stem Cell Institute Tissue Culture, University of Cambridge), 50 ng/mL recombinant human EGF (PeproTech, AF-100-15), 100 ng/mL recom-binant human Noggin (PeproTech, 120-10C), 100 ng/mL recombinant human FGF10 (PeproTech, 100-26), 100 ng/mL recombinant human FGF7 (PeproTech, 100-19), 3 μM CHIR99021 (Stem Cell Institute Tissue Culture, University of Cambridge) and 10 μM SB431542 (Bio-Techne, 1614)] in a CO_2_ incubator (balanced with air, 5% CO_2_) or hypoxia incubator (2-5% O_2_, 5% CO_2_). The medium was changed every 3 days. Any residual mesenchymal cells do not expand in the medium and are lost during passaging.^7^ Prior to use in experiments, organoids were inspected visually to ensure that no fibroblast cells were present. Organoids cultured under normoxia were passaged every 5-7 days depending on the confluence. For passaging organoids, fresh cold (4°C) AdvDMEM+++ was used to disrupt the BME mechanically and harvest organoids. The organoids were pelleted by centrifugation and dissociated using TrypLE (Thermo Fisher Scientific, 12605010) at 37°C for 10 min, or sheared by pipetting. The cells or organoid pieces were washed in AdvDMEM+++ and resuspended in BME according to subculture ratios. SRM was supplemented with 10 μM Y-27632 for first 3 days. Organoids cultured under hypoxia were passaged by mechanical shearing using a 200 μL pipette tip around every 2 weeks. For chemical treatment, Roxadustat (50 μM) and PT2385 (10 μM) were added to SRM and changed every 3 days. All fetal lung organoids tested negative for mycoplasma.

#### Derivation and maintenance of human fetal lung-derived AT2 (fdAT2) organoids

The dissection and isolation of distal epithelial cells from human second-trimester (17-21 pcw) lungs were as previously described.^9,45^ Briefly, the lung distal regions were cut into small pieces and dissociated in 5 mL of enzyme mixture (0.125 mg/mL Collagenase, Merck, C9891; 1 U/mL Dispase, Thermo Fisher Scientific, 17105041; 10 U/mL DNase, Merck, D4527) at 37°C for 1 hour with rotation. The cells were washed with AdvDMEM+++ medium and filtered through a 40 μm strainer. The supernatant was removed after centrifugation and the cell pellet was resuspended in red blood cell lysis buffer (BioLegend, 420301) for 5 min, and washed with AdvDMEM+++ medium. The dissociated cells were enriched for epithelial cells by Magnetic-activated cell sorting (MACS) (buffer: 1x PBS, 1% BSA, and 2 mM EDTA) with CD326 (EpCAM) microbeads (Miltenyi Biotec, 130-061-101) according to the manufacturer’s instructions. The enriched cells were resuspended in BME and seeded into multi-well plates for culture with the Alveolar Type 2 Medium (AT2M) [AdvDMEM+++, 1X B27 supplement (without Vitamin A), 1x N2 supplement, 1.25 mM n-Acetylcysteine, 10 mM CHIR99021, 50 μM Dexamethasone (Merck, D4902), 10 μM Y-27632, 0.1 M 8-Bromoadenosine 3’5’-cyclic monophosphate (cAMP; Merck, B5386), 0.1 M 3-Isobutyl-1-methylxanthine (IBMX; Merck, 15679), 50 mM DAPT (Merck, D5942), and 10 mM A83-01 (Tocris, 2939)]. Medium was changed every 3 days and the organoids were passaged around every 2 weeks. Alternatively, the dissociated cells were transduced by the *SFTPC-GFP* reporter lentiviral construct in suspension in AT2M overnight. The cells were collected and expanded in BME in AT2M for 5-6 days. The SFTPC-GFP^+^ cells were sorted (Sony SH800Z Cell Sorter) and cultured in AT2M. All fdAT2 lung organoids tested negative for mycoplasma.

#### Derivation and maintenance of human adult lung-derived AT2 (adAT2) organoids

Human adult lung parenchyma was dissected and dissociated as previously described.^51^ Briefly, human distal lung edges were cut into small pieces, and digested with 10 mL of enzyme mixture (Collagenase: 1.68 mg/mL, Dispase: 5 U/mL, DNase: 10 U/mL) at 37°C for 2-3h with rotation and pipetting in the middle to assist digestion. The cells were washed with AdvDMEM+++ medium and filtered through a 40μm strainer. The supernatant was removed after centrifugation at 500 g for 5 min and the cell pellet was resuspended in red blood cell lysis buffer (BioLegend, 420301) for 10 min, and washed with AdvDMEM+++ medium. Total cells were centrifuged at 500 g for 5 min and the cell pellet was processed by MACS with CD326 (EpCAM) microbeads (Miltenyi Biotec, 130-061-101) according to the manufacturer’s instructions. CD326 selected cells were further sorted with HTII-280 antibody (1:100, Terrace Biotech, TB-27AHT2-280) and goat anti-mouse IgM AF488 antibody (1:200, Thermo Fisher Scientific, A-21042) using BD FACSDiscover S8 Cell Sorter. Cells were monitored and imaged during sorting. Sorted HTII-280+ cells were resuspended in BME (5-10k cells per 30 uL BME drop) and seeded into multi-well plates for culture with AT2M or serum-free feeder-free (SFFF) medium (AdvDMEM+++, 1x B27 supplement (without Vitamin A), 1x N2 supplement, 1x ITS, 1.25 mM n-Acetylcysteine, 3 μM CHIR99021, 10 μM SB431542, 1 μM BIRB796, 50 ng/ml recombinant human EGF, 10 ng/ml recombinant human FGF10, 5 μg/ml Heparin, and 10 μM Y-27632) as previously reported.^51^ Medium was changed every 3 days and the organoids were passaged every 2-3 weeks. All adult lung organoids tested negative for mycoplasma.

#### Mouse breeding

Mice were bred and maintained under specific-pathogen-free conditions at the Gurdon Institute of the University of Cambridge. All mouse procedures were approved by the University of Cambridge Animal Welfare and Ethical Review Body and carried out under a UK Home Office License (PPL: PEEE9B8E4) in accordance with the Animals (Scientific Procedures) Act 1986.

#### Mouse embryonic lung dissection and tip progenitor organoid culture

The first day a vaginal plug was detected was designated as embryonic (E) day 0.5. The lungs of E11.5-E14.5 wild-type C57BL/6J mouse embryos were dissected. The lung buds were cut and briefly treated with Dispase (8 U/mL) to separate the mesenchyme. The epithelial tips were seeded in BME and cultured in adapted previously reported mouse lung tip progenitor medium (AdvDMEM+++, 1x ITS, 3 μM CHIR99021, 1 μM A83-01, 1 μM BIRB796, 50 ng/ml EGF, 50 ng/ml Fgf9, 50 ng/ml FGF10, 5 μg/ml Heparin, and 10 μM Y-27632).^30^ The organoids were passaged every 5-7 days using the same approaches as for human lung organoids.

### Method Details

#### Molecular cloning

For mutated HIF1α and HIF2α overexpression, the *HIF1A* and *HIF2A* CDS were cloned from plasmids gifted from William Kaelin (Addgene, #87261, #25956) and inserted into Tet-ON vectors with EF1a-TagRFP-2A-tet3G.^42,44^ For CRISPRi, the gRNA sequences targeting *HIF1A, HIF2A, KLF4, KLF5* were selected from a published database and inserted into U6-gRNA-EF1a-EGFP-CAAX lentiviral vectors (Addgene, #167936).^42,43^ For targeted DamID, the wild-type *HIF1A* and *HIF2A* CDS were inserted into DamID vectors with SFFV-mNeonGreen as upstream open reading frame.^28^ The tetON-KRAB-dCas9-DHFR-EF1a-TagRFP-2A-tet3G plasmid e6 Cell Stem Cell *32*, 1705–1722.e1–e9, November 6, 2025 (Addgene, #167935), NTC (non-targeting control) plasmid, *SFTPC-GFP* reporter plasmid and HRE-ODD-GFP reporter plasmid were as previously described.^9,39,42^

#### Lentiviral production and organoid transduction

HEK293T cells were grown in 10-cm dishes to 80% confluency before transfection with the lentiviral vector (10 μg) with packaging vectors including pMD2.G (3 μg, Addgene, # 12259), psPAX2 (6 μg, Addgene, #12260) and pAdVAntage (3 μg, Promega, E1711) using Lipofectamine 2000 Transfection Reagent (Thermo Fisher Scientific, 11668019) according to manufacturer’s protocol. After 16 hrs, medium was refreshed. Supernatant containing lentivirus was harvested at 24 hrs and 48 hrs after medium refreshing and pooled together. Supernatant was centrifuged to remove cell fragments and passed through 0.45 μm filter. The lentivirus was concentrated using AVANTI J-30I centrifuge (Beckman Coulter) or Lenti-X™ Concentrator (Takara, 631232) following the manufacturer’s protocol. For transduction, the organoids were dissociated by TrypLE and cultured in SRM with 10 μM Y-27632 in suspension with packaged viruses overnight. The cells were washed by AdvDMEM+++ and cultured in SRM with 10 μM Y-27632 for first 3 days. After 5-7 days, the organoids were dissociated for cell sorting with wild-type cells as the negative control. For Tet-ON overexpression, 2 μg/mL Doxycycline (Merck, D9891) was added into SRM. For CRISPRi, 2 μg/mL Doxycycline and 10 μM TMP (Merck, 92131) were used.

#### Airway and alveolar differentiation of first-trimester lung progenitors

After growing in SRM from single cells for 3 days, the first-trimester epithelial progenitor organoids were cultured in the Airway Differentiation Medium (AWDM) (AdvDMEM+++, 1X B27, 1X N2, 1.25 mMN-acetylcysteine, 100 ng/mL FGF10, 100 ng/mL FGF7, 50 nM Dexamethasone, 0.1 mM cAMP, 0.1 mM IBMX, 10 μM Y-27632), or the Alveolar Differentiation Medium (ADM) (AdvDMEM+++, 1X B27, 1X N2, 1.25 mM N-acetylcysteine, 10 mM CHIR99021, 50 mM DAPT, 10 μM SB431542, 50 nM Dexamethasone, 0.1 mM cAMP, 0.1 mM IBMX, 10 μM Y-27632) as previously described.^8,9^ Medium was changed every 3 days and organoids were differentiated for 9-15 days.

#### Immunohistochemistry

Human embryonic and fetal lungs were fixed at 4°C overnight in 4% (w/v) paraformaldehyde in PBS. Fixed lungs were washed in 15%, 20% and 30% (w/v) sucrose in PBS at 4°C for 1 hour and incubated in 1:1 (v/v) mixture of optimal cutting temperature compound (OCT, Tissue-tek, 4583):30% sucrose (in PBS) at 4°C overnight. The lungs were finally embedded and frozen in 100% OCT and stored at -70°C before sectioning. For immunostaining, fetal lung cryosections (10 μm) were washed in PBS and incubated in PBS with 0.3% Triton X-100 (0.3% PBTX) for 10 minutes. The sections were incubated in blocking buffer (1% bovine serum albumin, 5% normal donkey serum in 0.3% PBTX) at room temperature for 1 hour and incubated with primary antibodies (KLF4, 1:500, Proteintech, 11880-1-AP; KLF5, 1:500, Proteintech, 21017-1-AP; SOX9, 1:600, Merck, AB5535; TP63, 1:600, Cell Signaling Technology, 13109; E-cadherin, 1:1000, Thermo Fisher Scientific, 13-1900; TESC, 1:300, Proteintech, 11125-1-AP) at 4°C overnight. The sections were washed in PBS and incubated with secondary antibodies (donkey anti-rabbit 488, 1:1000, Invitrogen, A-21206; donkey antigoat 594, 1:1000, Invitrogen, A-11058; donkey anti-rat 647, 1:1000, Jackson Immunoresearch, 712-605-153) at room temperature for 2 hours. The sections were stained with DAPI (1 μg/mL) at room temperature for 20 minutes, washed and mounted in Fluoromount for imaging by Leica SP8 confocal microscope and Nikon AxR confocal microscope. Images were processed using Fiji (version 2.15.1).^71^

#### Organoid whole-mount immunostaining

The organoids were released from BME by washing in cold (4°C) AdvDMEM+++ medium and fixed in 4% PFA on ice for 30min. The organoids were then washed in PBS 3 times and incubated in 0.3% PBTX for 1 hour at 4°C. The organoids were blocked at 4°C overnight, followed by primary antibody incubation (SOX2, 1:500, Bio-techne, AF2018; SOX9, 1:500, Merck, AB5535; SOX9, 1:500, R&D Systems, AF3075; TP63, 1:400, Cell Signaling Technology, 13109; TP63, 1:400, R&D Systems, AF1916; KRT5, 1:500, BioLegend, 905901; SCGB3A2, 1:800, Abcam, ab181853; SCGB1A1, 1:800, Proteintech, 10490-1-AP; E-cadherin, 1:1000, Thermo Fisher Scientific, 13-1900; Fibronectin, R&D Systems; NKX2.1, 1:500, Abcam, ab76013; proSFTPC, 1:400, Merck, AB3786; proSFTPB, 1:400, Seven Hills, WRAB-55522; ZO1, 1:400, Invitrogen, 40-2200; ASCL1, 1:400, Abcam, EPR19840; Ki67, 1:500, Invitrogen, 14-5698-82; Laminin, 1:500, Abcam, ab11575; KRT13, 1:500, Abcam, ab79279; HIF1α, 1:300, Novus Biologicals, NB100-134; HIF2α, 1:300, Novus Biologicals, NB100-122; KLF4, 1:400, Proteintech, 11880-1-AP; KLF5, 1:400, Proteintech, 21017-1-AP; KRT8, 1:200, Santa Cruz, sc-8020; KRT17, 1:200, Santa Cruz, sc-393002; mature SFTPC, 1:300, Seven Hills, WRAB-76694; mature SFTPB, 1:300, Seven Hills, WRAB-48604; ABCA3, 1:200, Seven Hills, WRAB-ABCA3; TESC, 1:200, Proteintech, 11125-1-AP) at 4°C overnight. The organoids were washed in PBS and incubated in secondary antibodies (donkey anti-chicken 488, 1:1000, Jackson Immune, 703-545-155; donkey anti-rabbit 488, 1:1000, Invitrogen, A-21206; donkey anti-mouse 488, 1:1000, Invitrogen, A-21202; donkey anti-rat 488, 1:1000, Invitrogen, A-21208; donkey anti-mouse 594, 1:1000, Invitrogen, A-21203; donkey anti-rabbit 594, 1:1000, Invitrogen, A-21207; donkey anti-goat 594, 1:1000, Invitrogen, A-11058; donkey anti-sheep 594, 1:1000, Jackson Immunoresearch, 713-585-147; donkey anti-rat 647, 1:1000, Jackson Immunoresearch, 712-605-153; donkey anti-rabbit 647, 1:1000, Invitrogen, A-31573; donkey anti-mouse 647, 1:1000, Invitrogen, A-31571; donkey anti-goat 647, 1:1000, Invitrogen, A-21447) at 4°C overnight. After DAPI staining (1 μg/mL) at 4°C for 1 hour, the organoids were processed through a thiodiethanol series (25%, 50%, 75% and 97% v/v concentration in PBS) at 4°C followed by mounting in 97% thiodiethanol and imaging on Leica SP8 or Nikon AxR confocal microscopes. Images were processed using Fiji (version 2.15.1).^71^

#### Western blot

The organoid samples were harvested, lysed with RIPA buffer (Merck, R0278) after removing BME, and then run on 12.5% SDS-PAGE gels. Proteins were transferred onto PVDF membranes with BioRad Mini Trans-Blot system. The membranes were blocked with 5% skimmed milk in 0.1% Tween-20/TBS (TBST) for 30 minutes at room temperature, and incubated at 4°C overnight with primary antibodies (HIF1α, 1:1000, Novus Biologicals, NB100-134; HIF2α, 1:1000, Cell Signaling Technology, 7096; β-Actin, 1:5000, Merck, A1978) in 0.1% skimmed milk in TBST buffer (blocking buffer). After washing with TBST, the membranes were incubated with secondary antibodies conjugated with fluorescence dyes (anti-mouse IRDye® 800CW, 1:5000, Abcam, ab216774; anti-rabbit IRDye® 680RD, 1:5000, Abcam, ab216779) at room temperature for 3 hours. The membranes were washed with TBST and developed using the Li-Cor Odyssey imaging system.

#### RNA extraction, reverse transcription and RT-qPCR analysis

Organoids were harvested and the RNA was extracted using RNeasy Plus Mini Kit (Qiagen, 74134). The cDNA was synthesized using High-Capacity cDNA Reverse Transcription Kit (Thermo Fisher Scientific, 4368814). Incubation at 25 °C for 10 minutes, 37 °C for 2 hours and 85 °C for 5 minutes. For RT-qPCR, diluted cDNA was mixed with primers and PowerUp SYBR Green Master Mix (Thermo Fisher Scientific, A25741). Fold changes of target gene expression were determined by ΔΔCT methods with *ACTB* as reference gene. The primer sequence information is listed in Table S5. The data was analysed in GraphPad Prism 10 with one or two-way ANOVA with Tukey/Bonferroni/Dunnett multiple comparison tests or linear regression as stated in each figure. Significance levels: *p < 0.05, **p < 0.01, ***p < 0.001, ****p < 0.0001.

#### Bulk RNA-sequencing and analysis

The extracted RNA quality was analysed with High Sensitivity RNA ScreenTape (Agilent, 5067-5579) on Agilent 4200 Tapestation. The mRNA-sequencing library preparation and sequencing were completed by Novogene (UK) Company Limited with NovaSeq 6000. 20-50 M PE150 reads were sequenced for each sample. The sequencing data was analysed with nf-core/rnaseq pipeline (version 3.9) with default settings and the reads were mapped to human genome GRCh38.p13.^59^ The output gene count matrix was used for differential gene expression analysis with DESeq2.^60^ The differentially expressed genes (DEGs) were extracted by the contrast function by comparing hypoxia + NTC and normoxia + NTC, and hypoxia + HIF1α-knock down and hypoxia + NTC conditions separately. The DEGs (*Padj* < 0.05) were used for Gene Set Enrichment Analysis (GSEA) with Molecular Signatures Database (v2022.1.Hs).^29^

#### Organoid single-cell RNA sequencing and analysis

Lung progenitor organoids from two fetal lungs (9 pcw) cultured under normoxia and 8, 16, 24, and 32 days of hypoxia were harvested in parallel at each time point. The organoids were dissociated into single cells using TrypLE, filtered through a 40 μm filter to achieve > 90% single cells and evaluated by Trypan Blue Solution (Thermo Fisher Scientific, 15250061) to confirm > 90% cell viability. The cells were fixed and frozen using Evercode Cell Fixation kit (Parse Biosciences). All the samples were processed together with Evercode Whole Transcriptome v2 kit (Parse Biosciences) to generate sequencing libraries. The libraries were multiplexed and sequenced by BGI Group in one T7 lane to achieve an average of 63,000 raw PE reads per cell of estimated 82,391 total cells. The sequencing data was initially processed with split-pipe (Version 1.1.1, Parse Biosciences) to combine sublibraries. The reads were mapped to human genome GRCh38.p14. The gene count matrix was used for downstream analysis in Seurat (Version 5).^61^ The cells were filtered based on gene counts 2,000-7,000, RNA counts > 4,000, mitochondrial gene percentages < 5% and genes detected in > 100 cells to yield total 65,475 cells. Data was normalised (normalization.method = “LogNormalize”, scale.factor = 10000) and scaled with default settings. The linear dimensional reduction was based on top 2000 highly variable features. The cell clusters (using top 20 PCs, 13 neighbours, and resolution at 0.5) were curated and annotated based on canonical *in vivo* cell type markers as previously described to generate 11 cell types.^8,33^ The differentially expressed genes for each cell type were found using FindMarkers function using default parameters. The trajectory analysis was complemented with Monocle 3 (for both partitions containing tip and primed progenitors) and Slingshot (only the major partition containing primed progenitors) by setting the root at cycling cells.^37,38^ The cell cycle scoring was calculated with CellCycleScoring function in Seurat. For mapping the organoid data to a fetal lung epithelial cell atlas,^8^ the fetal lung epithelial cells were re-clustered with Seurat default settings (except dims = 1:50 in FindNeighbors) as the reference, and the organoid data projected onto the reference UMAP structure with FindTransferAnchors, TransferData, AddMetaData and MapQuery functions. The plots were created with DimPlot, FeaturePlot, DotPlot, and RidgePlot functions. The regulons were analysed with SCENIC on downsampled organoid data (500 cells for each of the 13 original clusters) in R.^62^ The DEGs between primed progenitors and normoxic tip progenitors, and between hypoxic tip progenitors and normoxic tip progenitors, were analysed with AggregateExpression pseudobulk function and FindMarkers function. The connect plots were based on GSEA results for the DEGs.

For fdAT2 organoids scRNA-seq experiment, fdAT2 organoids derived from one fetal lung (20 pcw) were cultured under normoxia, hypoxia (6, 15, and 30 days) and re-exposure to normoxia (6, 15, and 30 days). The harvested organoids were processed with the same methods above. The sequencing library was generated using Evercode Whole Transcriptome v3 mini kit (Parse Biosciences), and sequenced by Illumina NovaSeq X to achieve an average of 132,002 raw PE reads per cell of estimated 15,417 total cells. The sequencing data was processed with split-pipe (Version 1.5.0) and mapped to human genome GRCh38.p14. Downstream analysis was performed in Seurat (Version 5). The cells were filtered (gene counts 2,500-9,000, RNA counts < 60,000, mitochondrial gene percentages < 10% and genes detected in > 100 cells) to yield total 12,567 cells. The cells (top 20 PCs, resolution at 0.3) were clustered and analysed using the same functions and packages as for progenitor organoids scRNA-seq.

#### Spatial transcriptomic data analysis

The Spatial transcriptomic data of a fetal lung (15 post-gestational week) generated on the 10X Xenium platform was previously reported.^33^ The transcriptomes were clustered using the Louvain algorithm with resolution at 1, and were presented spatially. The image was zoomed to coordinates x = c(11600, 12000), y = c(5500, 5900) and plotted using Seurat v5, highlighting clusters 2 = “tip”, 5 = “stalk” and 16 = “Differentiating airway cells”. We used FindMarkers in Seurat with default parameters to identify differentially expressed genes in tip and stalk cells.

Spatial transcriptomic data from human fetal lungs (8, 9, and 10 pcw) generated on the 10x Visium platform were obtained from the Human Developmental Lung Cell Atlas.^31^ Data were processed in R 4.4.2 with Seurat v5. Spatially variable features were detected using FindVariableFeatures, then clustered by multilevel-refined Louvain. Spatial patterns including a significant number of HIF-DamID target genes were identified by chi-square testing (residual > 4). The gene ontology analysis was performed with Enrichr.^63^

#### Targeted DamID-sequencing sample preparation

The HIF1α, HIF2α and empty DamID-only lentiviral vectors were transduced to dissociated lung progenitor organoids from 3 donors as described above. 20-40% cells were transduced as checked by the mNeonGreen signals. The cells were cultured in SRM with 10 μM Y-27632 under normoxia for 3 days, and then treated with 2% O_2_ in SRM for 6 days. Medium was changed every 3 days. Then the organoids were harvested and processed for Illumina sequencing with an adapted TruSeq protocol as previously described.^41^ All samples were multiplexed and the sequencing was performed by the Cancer Research UK Cambridge Institute genomics facility using 1 lane of Illumina NovaSeq X as PE50 reads.

#### Targeted DamID-sequencing data analysis

Targeted DamID-sequencing data was processed using a publicly available Snakemake workflow.^64^ The reads were aligned to the human genome (Ensembl GRCh38.110) using bowtie2 v2.5.3.^65^ To prevent signals originating from the expression vectors of *Dam-HIF1A/HIF2A* fusion genes from obscuring the analysis, the bowtie2 index was built with a FASTA file where the genome sequences of *HIF1A* and *HIF2A* were masked. Subsequently, bedGraph files were generated with reads binned into fragments based on 5’-GATC-3’ sites and normalised to a separate Dam-only control sample of the same organoid line. The alignment and bedGraph generation steps were performed using damidseq_pipeline v1.5.3.^66^ The width of bins to use for mapping reads was set at 300. HIF1α and HIF2α bedGraph files from one organoid cell line (i.e. biological replicate) were quantile normalised against all the other organoids. For visualisation of individual loci, the logarithmic values in the bedGraph files were back-transformed. Average signal at individual loci was plotted with pyGenomeTracks v3.8.^67^ Broad peak calling was performed with the MACS2 v2.2.9.1 subcommand callpeak (broad-cutoff = 0.1 and q = 0.05) using bam files generated by damidseq_pipeline.^68^ Dam-HIF1α/HIF2α served as treatment samples and the Dam-only as control sample. Consensus peaks were identified only if peaks occurred in all three biological replicates with at least 1 bp overlap. Consensus peaks smaller than 100 bp were extended by 100 bp on both the 5’ and 3’ end. Consensus peaks were annotated to the nearest transcription start site (within 3kb) with the ChIPseeker v1.38.0 R package to find HIF1α and HIF2α target genes.^69^ Profile plots for HIF1α and HIF2α target genes were generated with deepTools v3.5.4.^70^ The gene ontology analysis for HIF1α and HIF2α common target genes was performed with Enrichr.^63^

### Quantification And Statistical Analysis

The number of replicates is provided in the figure legends. Data are expressed as average ± standard deviation (SD). Statistical analysis was performed in GraphPad Prism 10 using one or two-way ANOVA with Tukey/Bonferroni/Dunnett multiple comparison tests, or linear regression as stated in the figure legends. Definition of significance levels: *p < 0.05, **p < 0.01, ***p < 0.001, ****p < 0.0001.

## Supplementary Material

Supplemental information can be found online at https://doi.org/10.1016/j.stem.2025.09.007.

Supplemental information

## Data Availability

The information regarding sequencing data from this study and previous publications is attached in the key resources table. This paper does not report original code. Additional information required to reanalyze the data is available from the lead contact upon request.

## Highlights

- Hypoxia promotes airway, but inhibits alveolar, cell fates of human lung progenitors
- HIF1*α* and HIF2*α* have distinct targets and functions in lung epithelial development
- KLF4 and KLF5 drive basal and secretory cell differentiation downstream of HIFs
- Hypoxia induces aberrant airway differentiation of mature human AT2 cells

## In brief

Dong and colleagues uncover low oxygen-directed differentiation of human lung progenitors to airway rather than alveolar fate and show how the process is controlled by hypoxia-inducible factors. Hypoxia also converts differentiated alveolar cells into airway-like cells. Hypoxia is therefore both a developmental cue and a pathological factor in human lungs.
