# Supplementary material for: Hypoxia promotes airway differentiation in the human lung epithelium
Source: Cell Stem Cell. Author manuscript; Available in PMC 2025 Nov 16. (PMC7618356; doi:10.1016/j.stem.2025.09.007)
Supplement: Supplemental information [file EMS210609-supplement-Supplemental_information.zip › 1-s2.0-S1934590925003388-mmc1.pdf]

Cell Stem Cell, Volume *32*

Supplemental Information

Hypoxia promotes airway differentiation

in the human lung epithelium

Ziqi Dong, Niek Wit, Aastha Agarwal, Adam James Reid, Dnyanesh Dubal, Sina Beier, Krishnaa T. Mahbubani, Kourosh Saeb-Parsy, Jelle van den Ameele, James A. Nathan, and Emma L. Rawlins

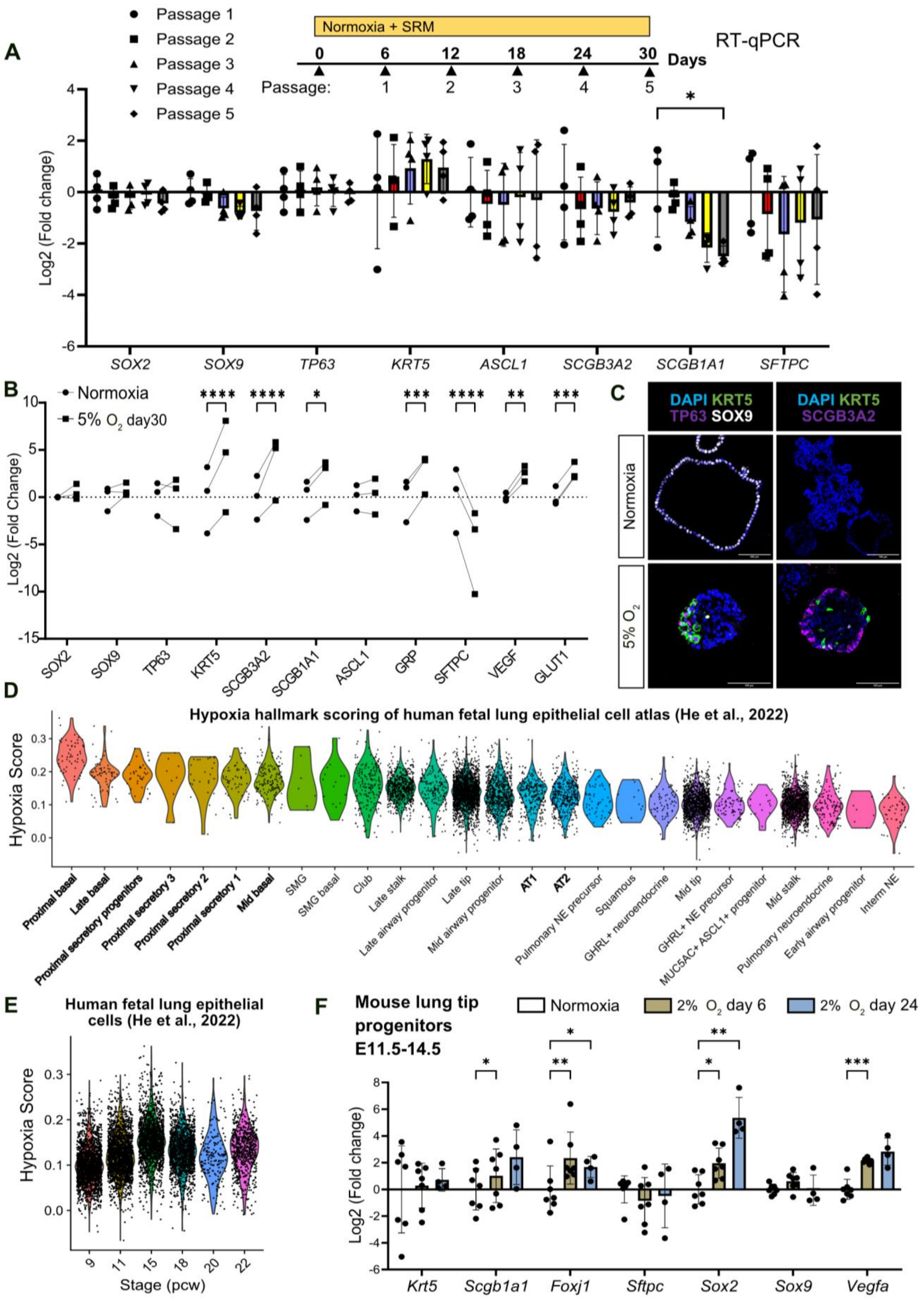

Figure S1. Hypoxia culture of human and mouse lung epithelial progenitors. Related to Figure 1.

(A) RT-qPCR of human lung progenitor organoids cultured under normoxia with routine passaging. Fold changes were normalised to the mean of Passage 1 organoids. Bar represents mean Log_2(fold change) ± SD, n = 4 biological donors. Statistical test: two-way ANOVA with Tukey's multiple comparisons test.

(B) RT-qPCR of human lung progenitor organoids cultured under normoxia or 5% O_2 for 30 days. Fold changes were normalised to the mean of the normoxia condition. Data shown as Log_2(fold change), n = 3 biological donors. Statistical test: two-way ANOVA with Bonferroni's multiple comparisons test.

(C) Immunostaining of human lung progenitor organoids cultured under normoxia or 5% O_2 for 30 days. Representative images of 2 organoid lines. Scale bars = 100 µm.

(D) and (E) Scoring the transcriptome of human fetal lung epithelial cells using the hypoxia hallmark gene list. Results visualised as cell types (D) or developmental stages (E).

(F) RT-qPCR of mouse lung progenitor organoids derived from E11.5-14.5 embryos cultured under normoxia or 2% O_2 for 6 and 24 days. Fold changes were normalised to the average of the normoxia condition. Bars represent mean Log_2(fold change) ± SD, n = 7 biological replicates. Statistical test: two-way ANOVA with Bonferroni's multiple comparisons test.

Gene expression was normalised to *ACTB* for RT-qPCR. Significance levels: $*p < 0.05$, $**p < 0.01$, $***p < 0.001$, $****p < 0.0001$.

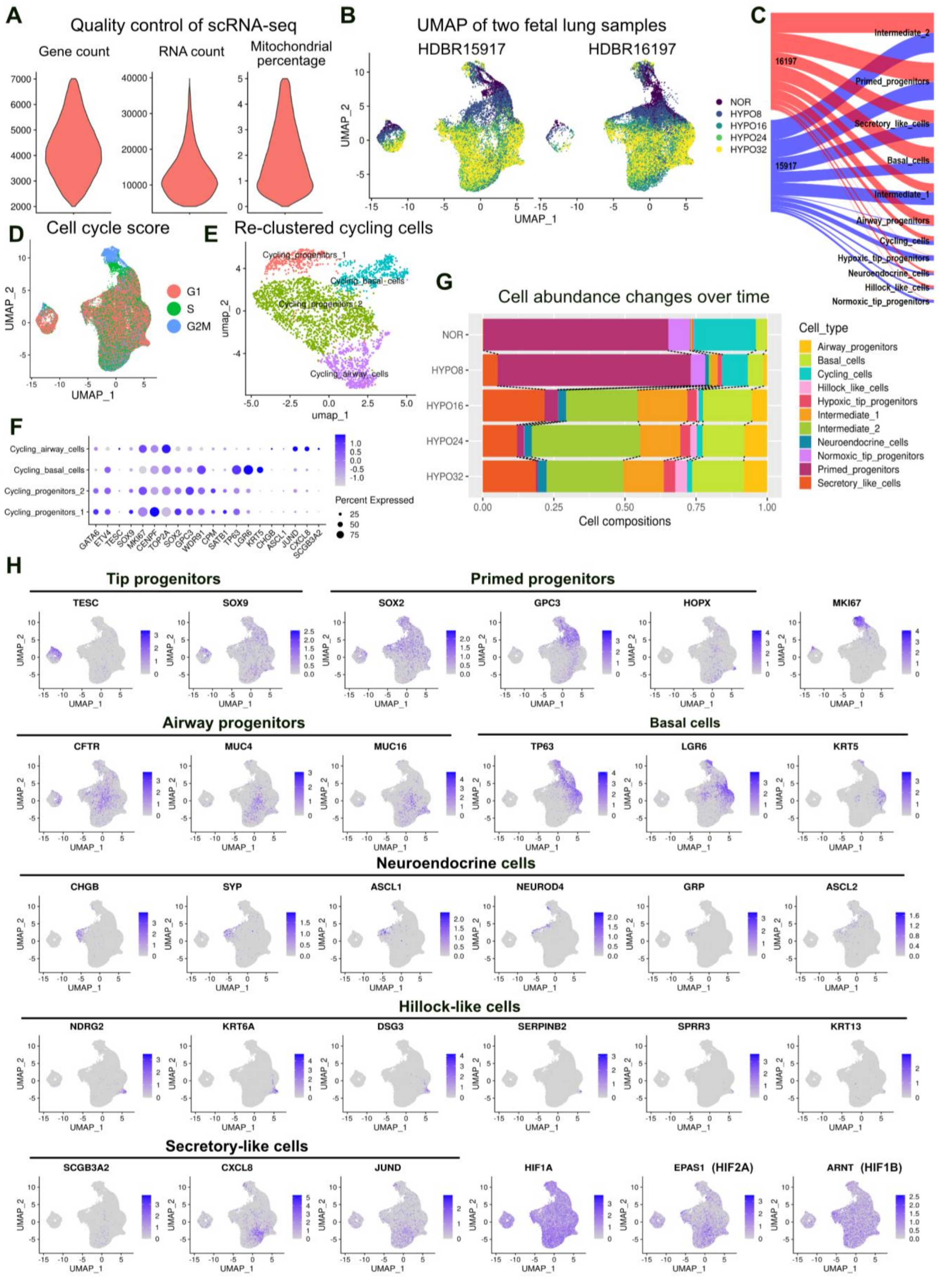

Figure S2. Characterisation of organoid single cell transcriptomic dataset. Related to Figure 2.

(A) Quality control and cell filtering standards, showing the gene count, RNA count and mitochondrial gene percentage for filtered cells.

(B) UMAP of cells sampled at different time points from two biological donors.

(C) The contribution of two biological donors to the annotated cell types.

(D) Cell cycle scores across all cells in the dataset.

(E) and (F) UMAP and marker gene expression of re-clustered cycling cells.

(G) Cell abundance changes of annotated cell types across different time points.

(H) Feature plots showing marker gene expression patterns. The plot for each gene was scaled to maximal expression level of the gene.

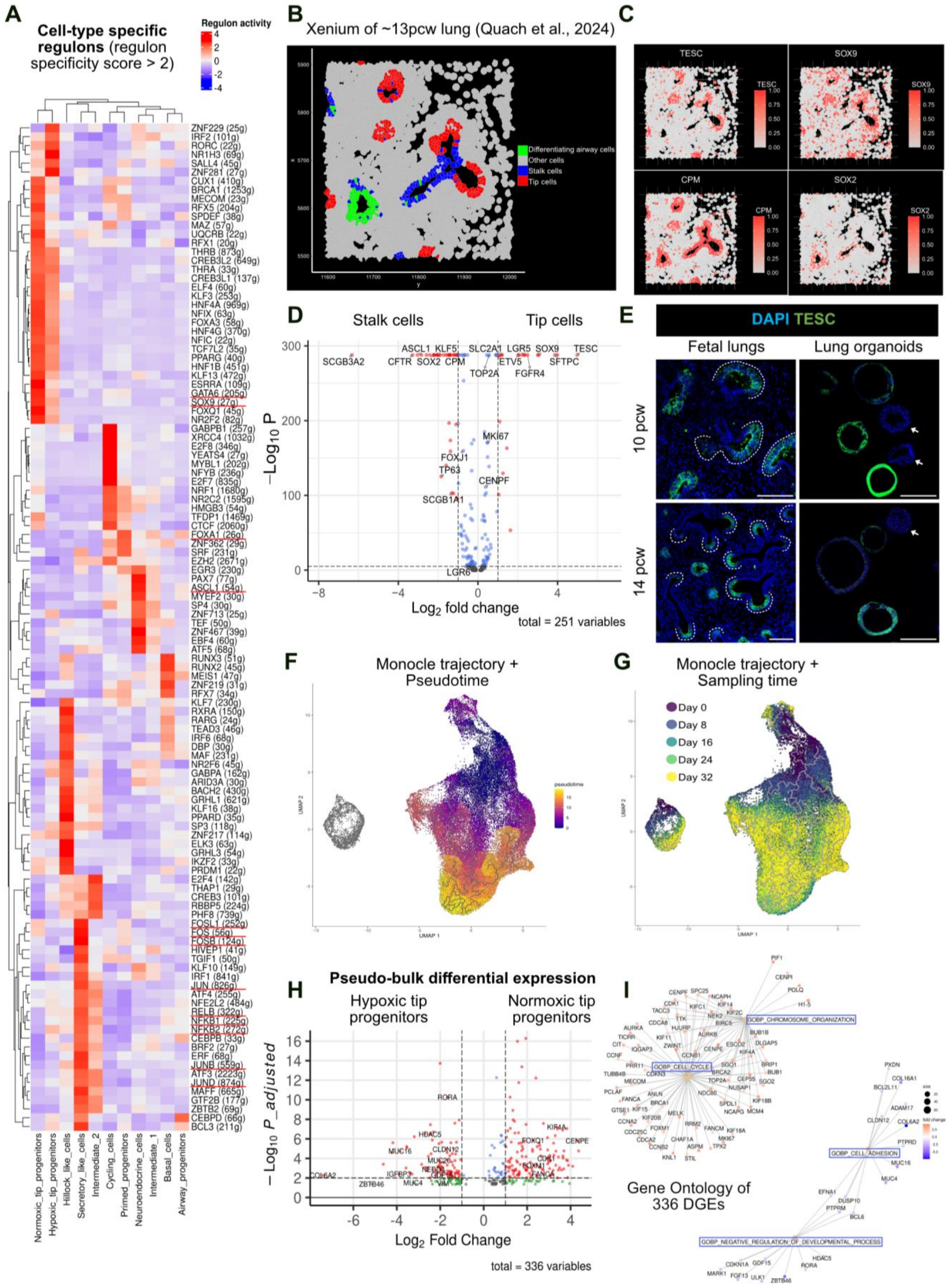

Figure S3. Regulons, trajectories, and differential gene expression analyses for organoid single cell transcriptomic dataset. Related to Figure 2.

(A) Cell-type specific regulons (filtered by regulon specificity score > 2) identified with SCENIC.

(B) Spatial visualisation of transcriptomic clustering of a human fetal lung Xenium dataset, highlighting clusters of tip cells, stalk cells, and differentiating airway cells.

(C) Expression patterns of *TESC*, *SOX9*, *SOX2*, and *CPM* in the Xenium data.

(D) Volcano plot of differentially expressed genes between tip and stalk cells in (B).

(E) TESC expression in human fetal lungs (10 and 14 pcw) and progenitor organoids derived from corresponding fetal lungs. The dash line indicates tip regions. The arrows indicate $TESC^{-}$ organoids. Scale bars = 100 μm.

(F) and (G) Monocle 3 trajectories overlaying with pseudotime (F) or actual sampling time (G).

(H) Volcano plot showing 336 filtered differentially expressed genes (*Padj* < 0.05) between hypoxic tip progenitors and normoxic tip progenitors generated by pseudobulk analysis with DESeq2.

(I) Linkage between DEGs in (H) and top-ranked Gene Ontology terms.

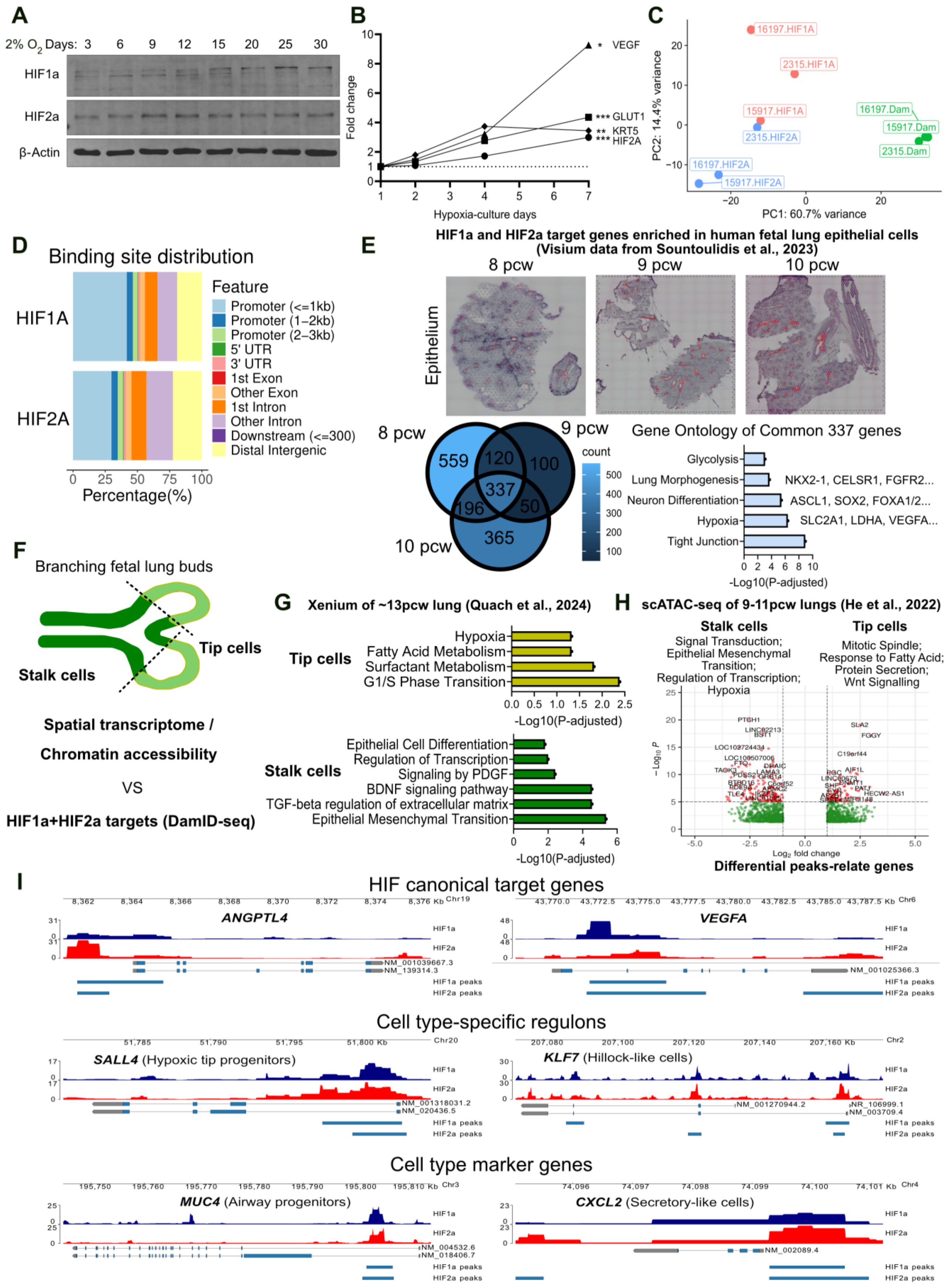

Figure S4. Activation of the HIF pathway and analyses of HIF1α and HIF2α targeted DamID-seq. Related to Figure 3.

(A) Protein expression level of HIF1α, HIF2α and β-actin detected by western blot in organoids cultured under hypoxia for 3-30 days.

(B) RT-qPCR of organoids cultured under hypoxia for 1, 2, 4, 7 days. Fold changes were normalised to hypoxia day 1 expression levels. Data shown as mean fold change, n = 3 biological donors. The significance of the curve slopes differing from zero was tested by linear regression. Gene expression was normalised to *ACTB*. Significance levels: *p < 0.05, **p < 0.01, ***p < 0.001.

(C) PCA plot of DamID samples from 3 organoid lines.

(D) Genomic categories of HIF1α and HIF2α binding sites.

(E) Visualisation and analysis of HIF (HIF1α and HIF2α) target genes enriched in the fetal lung epithelium. Representative images with labelled epithelium-enriched gene clusters shown for three fetal lungs (8, 9, and 10 pcw) from a Visium dataset. Venn diagram showing subsets of HIF target genes enriched in the lung epithelium at different developmental stages. Gene ontology analysis was performed for the commonly expressed 337 genes. Complete gene lists and gene ontology analysis in Table S5.

(F) Comparing HIF (HIF1α and HIF2α) target genes with the differentially expressed genes (DEGs) identified from a Xenium spatial transcriptome dataset, and differentially accessible regions identified from a scATAC-seq dataset, of tip and stalk cells.

(G) Gene ontology analysis of the overlapping genes between HIF targets and DEGs of tip and stalk cells identified from published Xenium data. Complete gene lists and gene ontology analysis in Table S5.

(H) Volcano plot and gene ontology analysis of the overlapping genes between HIF targets and differential ATAC-seq peak-related genes. The tip and stalk cells (9-11 pcw) were defined in a scATAC-seq dataset. Complete gene lists and gene ontology analysis in Table S5.

(I) Gene track views showing averaged DamID signals from three biological replicates over representative HIF1α and HIF2α target genes with consensus peaks labelled. The cell type markers and regulon transcription factors were selected from the organoid scRNA-seq dataset.

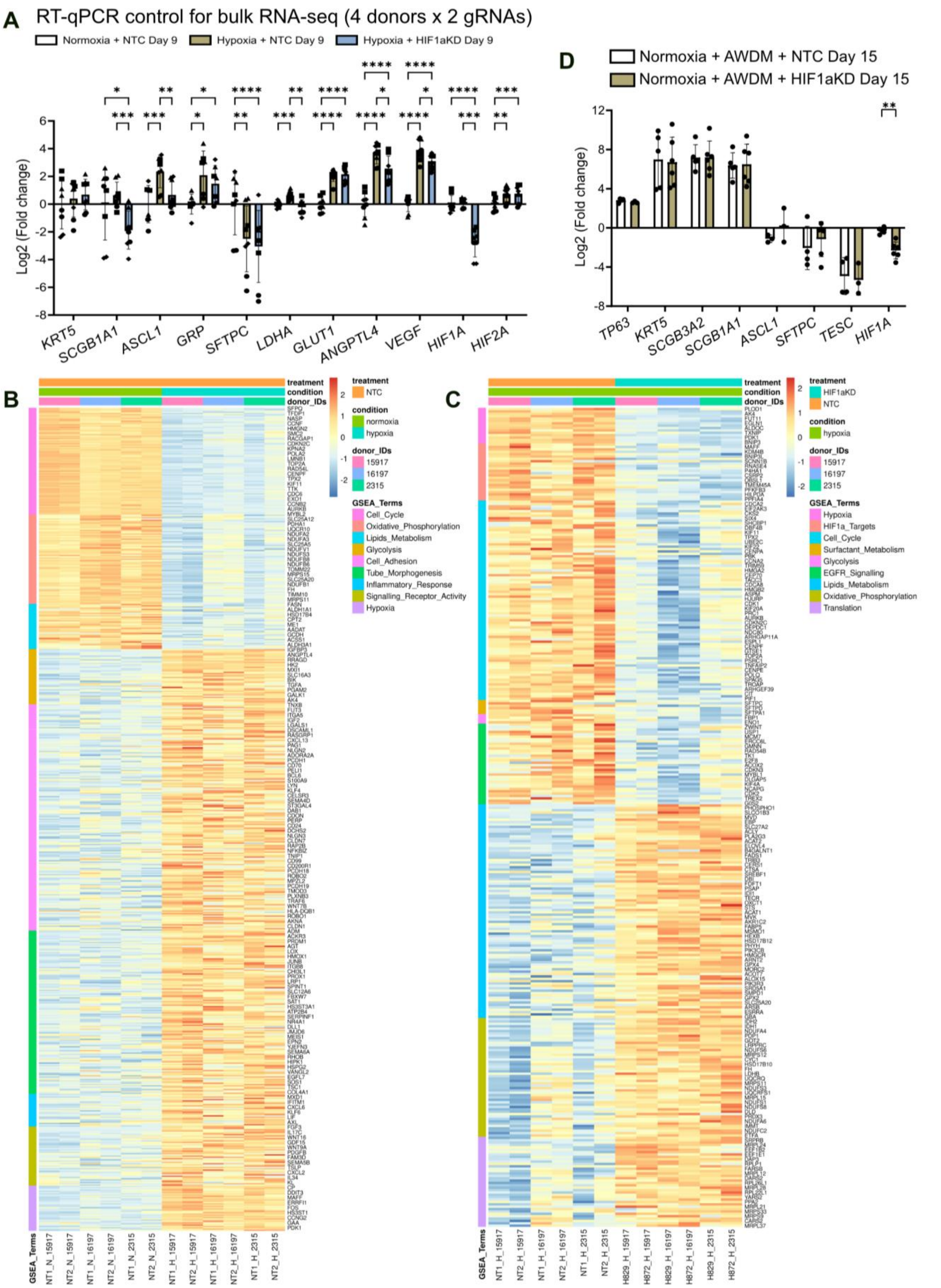

Figure S5. Bulk RNA-seq, differential gene expression analysis and airway differentiation tests for HIF1α-CRISPRi organoids. Related to Figure 4.

(A) RT-qPCR control of samples used for bulk RNA-seq. NTC and *HIF1A*-knock down (*HIF1A*-KD) organoids were cultured under normoxia or hypoxia for 9 days. Fold changes were normalised to the mean of NTC + normoxia condition. Bars represent mean Log_2(fold change) ± SD, n = 8 experimental replicates from

4 biological donors with 2 gRNAs. For bulk RNA-seq, 6 replicates (3 biological donors with 2 gRNAs) for each condition were selected. Statistical test: two-way ANOVA with Tukey's multiple comparisons test.

(B) Heatmap of 655 DEGs related to GSEA terms enriched in hypoxia compared to normoxia NTC organoids. Every 1 in 4 genes are labelled due to space limitations.

(C) Heatmap of 345 DEGs related to GSEA terms enriched in *HIF1A*-KD compared to NTC hypoxic organoids. Every 1 in 2 genes were labelled due to space limitations.

(D) RT-qPCR of NTC and *HIF1A*-KD organoids cultured in airway differentiation medium (AWDM) under normoxia for 15 days. The fold changes were normalised to the mean of normoxia + SRM condition (not shown). Bars represent Log_2(fold change) ± SD, n = 5 (NTC), 6 (*HIF1A*-KD) experimental replicates from 3 biological donors with 2 gRNAs. Statistical test: two-way ANOVA with Bonferroni's multiple comparisons test.

Gene expression was normalised by *ACTB* in RT-qPCR. Significance levels: *$p < 0.05$, **$p < 0.01$, ***$p < 0.001$.

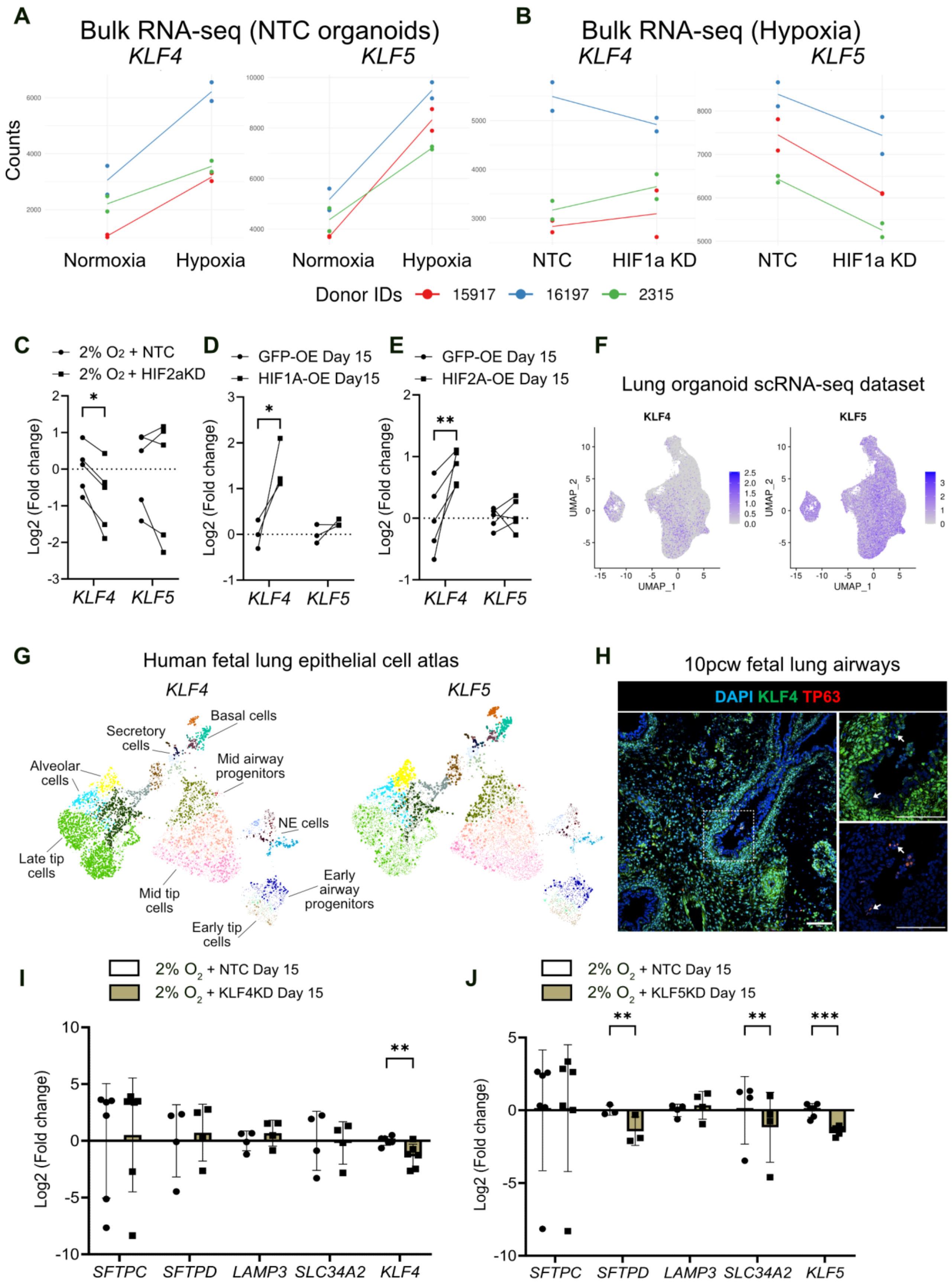

Figure S6. Expression patterns and hypoxic regulation of KLF4 and KLF5. Related to Figure 6.

(A) and (B) *KLF4* and *KL5* expression level from bulk RNA-seq data (as described in Figure 4) comparing Normoxia + NTC to Hypoxia + NTC (A), or Hypoxia + NTC to Hypoxia + *HIF1A*-knock down (KD) (B), n = 6 (2 gRNAs and 3 donors) for each condition. Each line indicates the change of the average counts of the two gRNA replicates from the same biological donor.

(C) *HIF2A* knock down decreased *KLF4* but not *KLF5* expression under hypoxia. Data shown as Log_2(fold change), n = 5 experimental replicates from 4 biological donors. 2 gRNAs used.

(D) and (E) Stabilised HIF1α and HIF2α (as described in Figures 4,5) overexpression under normoxia increased *KLF4* expression. Data shown as Log_2(fold change), n = 3 (HIF1α), 5 (HIF2α) biological donors.

(F) *KLF4* and *KLF5* expression in organoid scRNA-seq dataset (as described in Figure 2).

(G) *KLF4* and *KLF5* expression in epithelial cells of the human fetal lung atlas.

(H) Immunostaining of 10 pcw human fetal lung section showing KLF4 and TP63 expression. Arrows indicate $KLF4^{+}TP63^{+}$ cells. Scale bars = 100 µm.

(I) and (J) RT-qPCR detection of AT2 markers genes in NTC and *KLF4* or *KLF5*-CRISPRi organoids. Data shown as Log_2(fold change), n = 6 experimental replicates from 4 biological donors. 2 gRNAs used for each gene.

RT-qPCR gene expression was normalised to *ACTB*. Statistical test: two-way ANOVA with Bonferroni's multiple comparisons test. Significance levels: $*p < 0.05$, $**p < 0.01$, $***p < 0.001$.

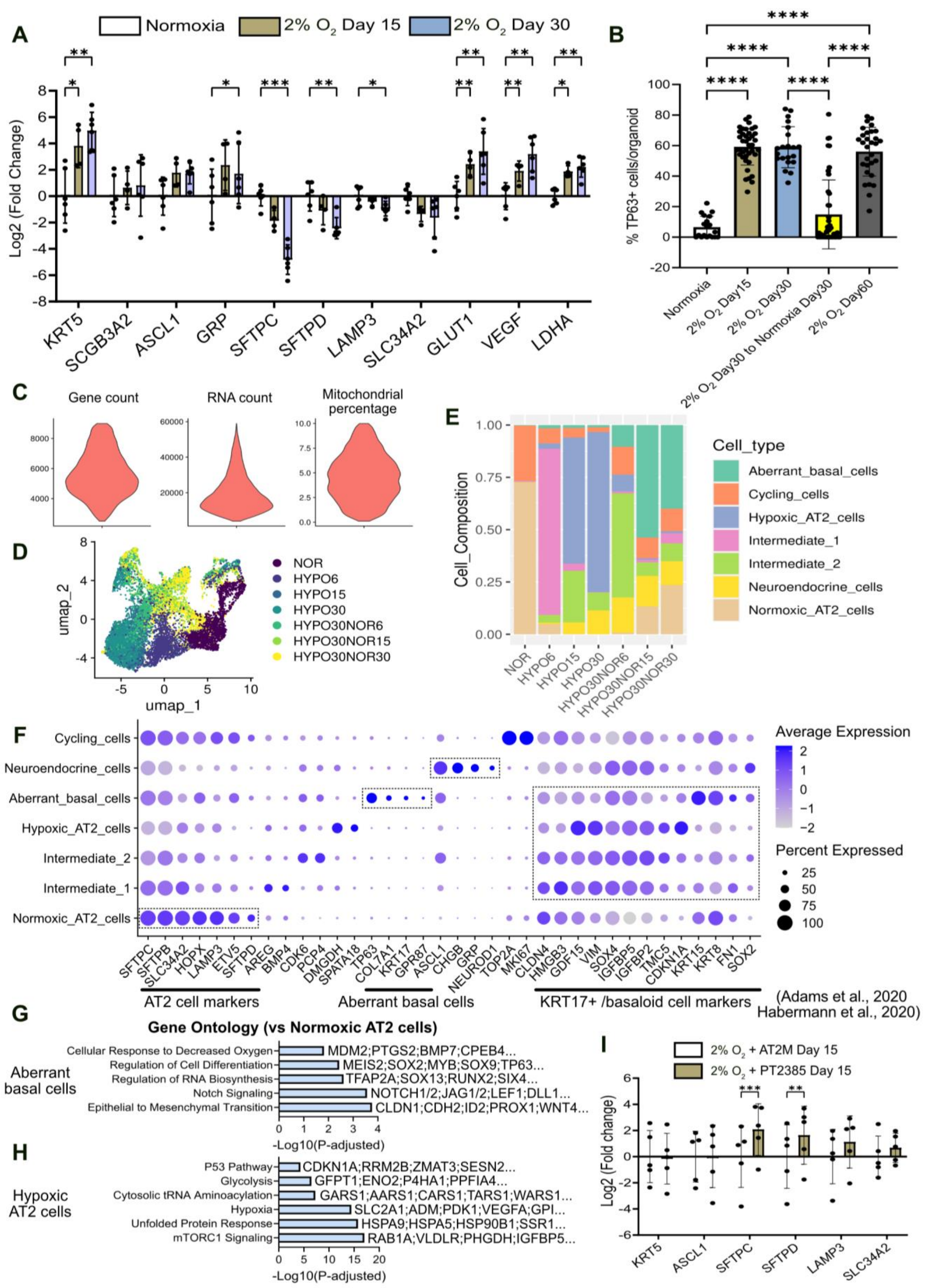

Figure S7. Analysis of hypoxia-treated fdAT2 organoids. Related to Figure 7.

(A) RT-qPCR of fdAT2 organoids cultured under normoxia or hypoxia for 15 and 30 days. Fold changes were normalised to the mean of the normoxia condition. Data shown as mean Log_2(fold change) ± SD, n = 6 experimental replicates from 4 biological donors.

(B) Quantification of $TP63^+$ cells/organoid based on immunostaining images acquired from 3 fdAT2 organoid lines. Each dot represents data from one organoid.

(C) Quality control of fdAT2 organoids scRNA-seq dataset, showing the gene count, RNA count and mitochondrial gene percentage for filtered cells.

(D) UMAP of fdAT2 organoids dataset showing sampling time points.

(E) Cell abundance changes of annotated cell types in fdAT2 organoids across different time points.

(F) Expression patterns of cell type-specific markers, and $KRT5^-KRT17^+$/basaloid cell markers identified from human IPF lung atlases.

(G) and (H) Gene ontology analysis of differentially expressed genes enriched in aberrant basal cells (196 genes) (G) and hypoxic AT2 cells (404 genes) (H), compared to normoxic AT2 cells. The differentially expressed genes were derived by pseudobulk analysis using DEseq2 and filtered by *P*-adjusted < 0.05.

(I) RT-qPCR of fdAT2 organoids treated with PT2385 or AT2M (with DMSO) under hypoxia for 15 days. Fold changes were normalised to the mean of the control condition. Data shown as mean Log_2(fold change) ± SD, n = 5 experimental replicates from 4 biological donors.

Gene expression was normalised to *ACTB* in RT-qPCR. Statistical test: two-way ANOVA with Bonferroni's multiple comparisons test. Significance levels: *p < 0.05, **p < 0.01, ***p < 0.001.

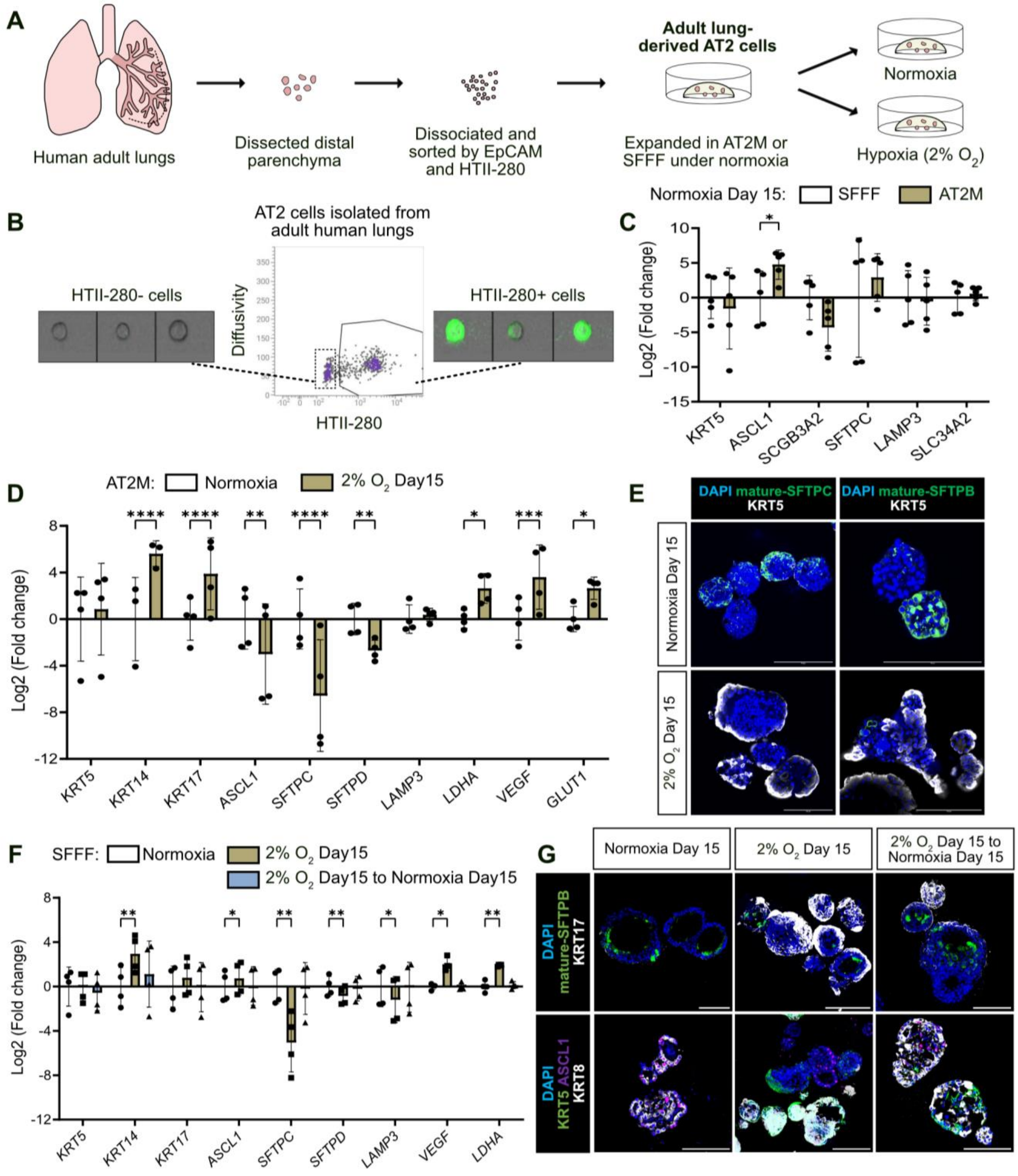

Figure S8. Derivation, culture, and hypoxia treatment of adAT2 organoids. Related to Figure 7.

(A) Experimental design for the derivation and culture of adult lung-derived AT2 organoids.

(B) FACS sorting adAT2 cells using the HTII-280 antibody.

(C) Comparing gene expression for adAT2 organoids cultured in SFFF and AT2M under normoxia. Fold changes were normalised to the mean of SFFF condition. Data shown as mean Log_2(fold change) ± SD, n = 5 biological donors.

(D) RT-qPCR of adAT2 organoids cultured in AT2M under normoxia or hypoxia for 15 days. Fold changes were normalised to the mean of the normoxia condition. Data shown as mean Log_2(fold change) ± SD, n = 4 biological donors.

(E) Immunostaining of adAT2 organoids cultured in AT2M showing decrease of mature-SFTPC and mature-SFTPB, and appearance of $KRT5^{+}$ cells in hypoxia. Representative images of 2 organoid lines. Scale bars = 100 μm.

(F) RT-qPCR of adAT2 organoids cultured in SFFF medium under normoxia, hypoxia for 15 days, and re-exposure to normoxia for 15 days. Fold changes were normalised to the mean of the normoxia condition. Data shown as mean Log_2(fold change) ± SD, n = 4 biological donors.

(G) Immunostaining of adAT2 organoids cultured in SFFF medium showing effects of hypoxia and re-exposure to normoxia on AT2 and airway cell markers. Representative images of 2 organoid lines. Scale bars = 100 μm.

Gene expression was normalised to *ACTB* in RT-qPCR. Statistical test: two-way ANOVA with Bonferroni's multiple comparisons test. Significance levels: *p < 0.05, **p < 0.01, ***p < 0.001, ****p < 0.0001.
